# Lactobacillus maintains healthy gut mucosa by producing L-Ornithine

**DOI:** 10.1038/s42003-019-0424-4

**Published:** 2019-05-08

**Authors:** Houbao Qi, Yuanyuan Li, Huan Yun, Tong Zhang, Yugang Huang, Jiang Zhou, Hui Yan, Jianmei Wei, Yingquan Liu, Zhiqian Zhang, Yunhuan Gao, Yongzhe Che, Xiaomin Su, Dashuai Zhu, Yuan Zhang, Jin Zhong, Rongcun Yang

**Affiliations:** 1 State Key Laboratory of Medicinal Chemical Biology, Nankai University, 300071 Tianjin, China; 2 Key Laboratory of Bioactive Materials Ministry of Education, Nankai University, 300071 Tianjin, China; 3 Department of Immunology, School of Medicine, Nankai University, 300071 Tianjin, China; 4 State Key Laboratory of Microbial Resources, Institute of Microbiology, Chinese Academy of Sciences, 100101 Beijing, China; 5 School of Life Science, University of Chinese Academy of Sciences, 100039 Beijing, China

**Keywords:** Bacterial host response, Mucosal immunology

## Abstract

Gut mucosal layers are crucial in maintaining the gut barrier function. Gut microbiota regulate homeostasis of gut mucosal layer via gut immune cells such as RORγt (+) IL-22(+) ILC3 cells, which can influence the proliferation of mucosal cells and the production of mucin. However, it is unclear how gut microbiota execute this regulation. Here we show that lactobacilli promote gut mucosal formation by producing L-Ornithine from arginine. L-Ornithine increases the level of aryl hydrocarbon receptor ligand L-kynurenine produced from tryptophan metabolism in gut epithelial cells, which in turn increases RORγt (+)IL-22(+) ILC3 cells. Human REG3A transgenic mice show an increased proportion of L-Ornithine producing lactobacilli in the gut contents, suggesting that gut epithelial REG3A favors the expansion of L-Ornithine producing lactobacilli. Our study implicates the importance of a crosstalk between arginine metabolism in Lactobacilli and tryptophan metabolism in gut epithelial cells in maintaining gut barrier.

## Abstract (web summary)

Qi, Li, Yun, Zhang et al.show that *lactobacilli* promote the generation of gut mucosa by facilitating the conversion of arginine to L-Ornithine. This study highlights the importance of a crosstalk between arginine metabolism in *Lactobacilli* and tryptophan metabolism in gut epithelial cells in maintaining a healthy gut barrier.

## Introduction

Gut mucus layers play a crucial barrier role in both separating the host from the noxious external environment and inhibiting the entrance of gut microbiota and/or their metabolites into the bloodstream and tissues^1^. The small intestine has one layer of unattached mucus to directly form a soluble mucus gel^2^, which may act as a matrix to limit the contact of gut microbiota with gut cell surface^3^. The colonic mucus layer forms a physical barrier against bacteria and their metabolites^4^. Although gut mucus layers are vitally important for individual health, the mechanism(s) underlying the maintenance of gut mucosal homeostasis is not completely clear.

Gut mucus layer consists of high-molecular-weight glycoproteins called mucin, that are synthesized and secreted by goblet cells. Goblet cells originate by their own mitosis or by differentiation of stem cells^5^, which may be regulated by gut immune cells through the production of cytokines, such as IL-6^6^ or direct cell–cell contact by activated macrophages^7^. IL-22 produced by innate lymphoid cells group 3 and other immune cells such as Th17, Th22, natural killer cells, γδ T cells, and lymphoid tissue inducer, can also promote the production of gut epithelial stem cells, which potentially increase mucus production through goblet cells^8,9^. These immune cell responses are dictated not only via “endogenous” host-derived but also “exogenous” signals, such as gut microbiota/their metabolites. Indeed, gut microbiota may not only regulate gut innate immune but also adaptive immune cells, such as that *L. reuteri* has a role in IL-22 production^10^, and segmented filamentous bacteria may induce Th17 cells differentiation^11^. The products of bacteria may also interrupt T-cell differentiation, such as that polysaccharide A from *Bacteroides fragilis* promotes Treg cell secretion^12^, and the lysates of polysaccharide-producing bacteria induce differentiation of Treg cells and IL-10 production^13^. Thus, the altered gut microbiota has direct or indirect effects on the gut immune cells.

Interestingly, many secreted antimicrobial proteins in the gastrointestinal tract may potentially affect the composition of gut microbiota via killing bacteria, such as REG3^14^. Recent studies have shown that Reg3α contributes to resistance to DSS-mediated colitis with altered gut microbiota^15^. Thus, it is possible for gut antimicrobial proteins to be involved in gut mucosal homeostasis through the altered microbiota. We here found that gut antimicrobial protein REG3A may affect the composition of gut microbiota, typically increasing the proportion of *Lactobacillus*. We demonstrate that these increased *Lactobacillus* may promote gut mucus-layer homeostasis through producing L-Orn.

## Results

### REG3A promotes the formation of gut mucus layers

**Fig. 1:**
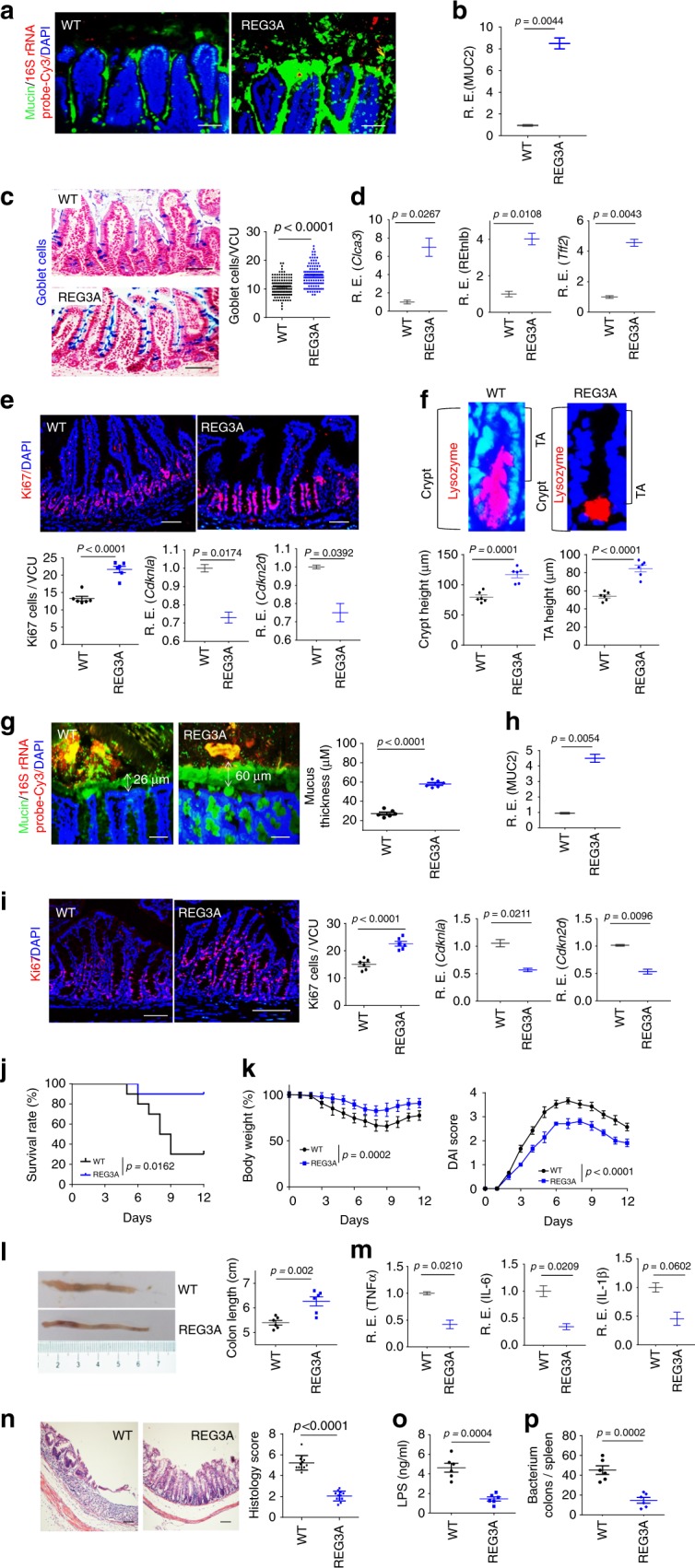
Gut human REG3A promotes the formation of gut mucus layers. **a** Fluorescence in situ hybridization of 16S rRNA and immunostaining of mucin in the ileum of human *REG3A*^*tg*^ mice (REG3A) and control cohoused littermate *wt* mice (ten slides/mouse; *n* = 6). **b** qRT-PCR of mucin 2 (MUC2) in the ileum of human *REG3A*^*tg*^ and control cohoused littermate wt mice (*n* = 6). **c** Staining of goblet cells in the ileum of control cohoused littermate *wt* and human *REG3A*^*tg*^ mice. Ten slides/mouse, *n* = 6; VCU, villus-crypt units. **d** QRT-PCR of Clca3, REtnlb, and Tff2 (*n* = 6). **e** Staining of Ki67 cells (ten slides/mouse, *n* = 6) and qRT-PCR of Cdknla and Cdkn2d (*n* = 6). **f** Crypt and transit-amplifying (TA) heights in the ileum of wt and human *REG3A*^*tg*^ mice. Eighty wt (WT) versus 86 human *REG3A tg* (REG3A) transit-amplifying compartments; ten slides/mouse, *n* = 6. **g** Fluorescence in situ hybridization of 16S rRNA and immunostaining of mucin in the proximal colon of human *REG3A*^*tg*^ mice (REG3A) and control cohoused littermate wt mice (ten slides/mouse; *n* = 6). **h** QRT-PCR of mucin 2 (MUC2) in the colon tissues (*n* = 6). **i** Staining of Ki67 cells in the colon (ten slides/mouse, *n* = 6) and qRT-PCR of Cdknla and Cdkn2d (*n* = 6). **j**, **k** Survival rate (**j**), body weight, and the disease activity index (DAI) (**k**) after DSS (*n* = 18). **l** Length of colon tissue. **m** QRT-PCR of TNFα, IL1^®^, and IL-6 in the colon tissues after DSS (*n* = 6). **n** Hematoxylin/eosin staining and histological scores of distal colon samples after DSS. Scale bars = 40 µm. **o** LPS in the peripheral sera of *REG3A*^*tg*^ and control cohoused littermate wt mice after DSS (*n* = 6). **p** Bacterium clones in the spleen after DSS (*n* = 6). Student’s *t* test, mean ± SD in **b**, **d**, and **e** (RE), **h**, **i** (RE), and **l**, **m**, **o**, and **p**, mean ± SEM in e (ki67 cell), **f**, **g**, and **i** the Mann–Whitney U test in **c** and **n**; Wilcoxon’s test in **j**; analysis of variance test in **k**; NS no significance; RE relative expression. Data are representative of three independent experiments. Also see Supplementary Figs. 1 and 2

To investigate the effect(s) of gut microbiota on gut mucosal-layer homeostasis, we generated human *REG3A*^*tg*^ mice, which may affect the composition of gut microbiota. We found that mucus gel remarkably increased in the ileum of *REG3A*^*tg*^ mice (Fig. 1a), in which human REG3A (Gene ID: 5068) was selectively expressed in mouse intestinal Paneth cells under the control of the HD5 promoter^16^ (Supplementary Fig. 1a, c, e). Higher levels of mucin 2 were also detected in the ileum of *REG3A*^*tg*^ mice (Fig. 1b). Intestinal histological structures of *REG3A*^*tg*^ mice exhibited increased goblet cells (Fig. 1c). The goblet cell markers *Clca3* (Gob5), *Retnlb* (RELMβ), and *Tff2* (trefoil factor 2) were upregulated in these *REG3A*^*tg*^ mice (Fig. 1d). Ki67 cells in the intestinal crypt also remarkably increased in *REG3A*^*tg*^ mice (Fig. 1e). The cell-cycle checkpoint molecules *Cdkn1a* (p21) and *Cdkn2d* (p19) were downregulated in these epithelial cells (Fig. 1e). Meanwhile, increased crypt height, including the transit-amplifying compartment, which indicates high proliferative activity, was also observed in these human *REG3A*^*tg*^ mice (Fig. 1f). Interestingly, mucus layers in the proximal colon tissues of *REG3A*^*tg*^ mice were also markedly thicker, as compared with their control cohoused littermates (Fig. 1g). The thickened mucus layer in the colon tissues may be derived from the expression of REG3A in colon Paneth cell-like cells^17^ and/or the secreted REG3A by intestinal Paneth cells. Higher levels of mucin 2 were detected in proximal colon tissues of human *REG3A*^*tg*^ mice (Fig. 1h). Ki67 cells in the colon crypt also remarkably increased in these *REG3A* ^*tg*^ mice (Fig. 1i). The *Cdkn1a* (p21) and *Cdkn2d* (p19) were downregulated in the colonic epithelial cells (Fig. 1i). The *REG3A*^*tg*^ mice also conferred a marked resistance to DSS-mediated colitis (Fig. 1i–n). Levels of serum LPS were lower in DSS-treated human *REG3A*^*tg*^ mice (Fig. 1o). The bacterium numbers in the organs and tissues, such as the spleen of DSS-treated *REG3A*^*tg*^ mice, were much less than wt control littermates (Fig. 1p). Furthermore, there had been much more goblet cells and Ki67 cells with upregulated *Clca3*, *Retnlb*, and *Tff2* and downregulated *Cdkn1a* and *Cdkn2d* in the colon crypt of DSS-treated human *REG3A*^*tg*^ mice (Supplementary Fig. 2a–d). Taken together, these data indicate that REG3A is involved in the maintenance of gut mucosal homeostasis through modulating gut epithelial regeneration and repair.

### REG3A-mediated formation of gut mucus layers is dependent on ILC3

**Fig. 2:**
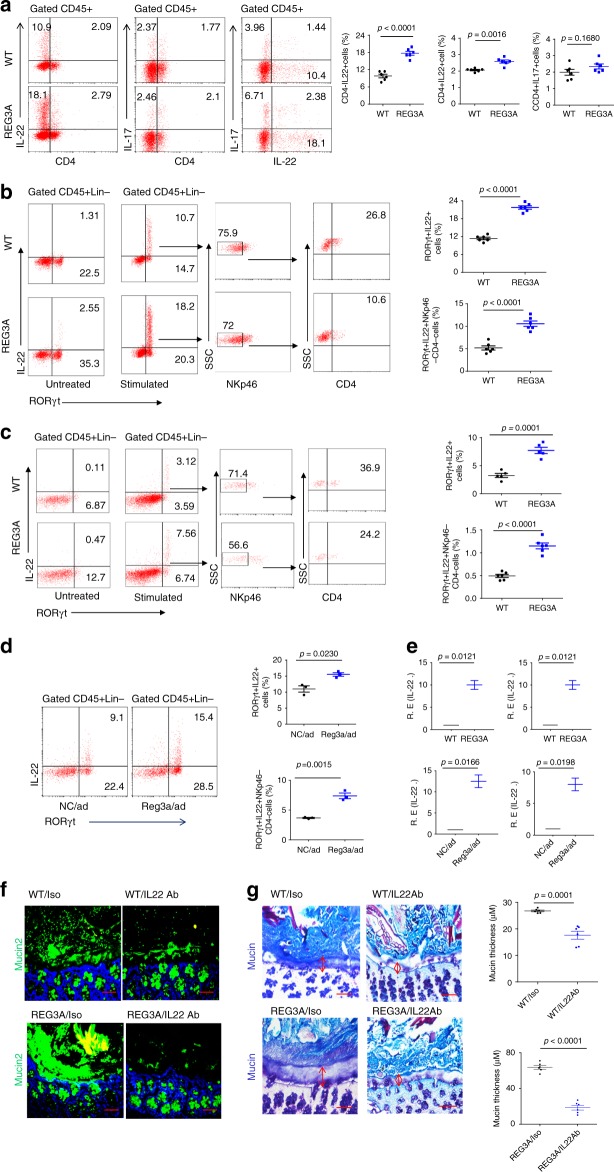
REG3A-mediated gut mucus layers depend on RORγt (+)IL-22 (+) ILC3 cells. **a** Flow cytometry of CD4(+)IL22(+), CD4(–)IL-22(+), CD4(+)Th17(+), and Th22(+)IL17(–) cells in the ileum lamina propria (LP) of human *REG3A*^*tg*^ (REG3A) and control cohoused littermate wt mice (*n* = 6). **b**, **c**, Flow cytometry of ROR^©^t(+)IL22(+) cells and their subsets in the ileum (**b**) and colon (**c**) LP of wt and human *REG3A*^*tg*^ mice (*n* = 6). **d** Flow cytometry of ROR^©^t(+)IL22(+) cells in the ileum LP of mice with (Reg3〈/ad) or without (NC/ad) Reg3〈/adenovirus injection (*n* = 3). **e** QRT-PCR of IL-22 in the ileum and colon of human *REG3A*^*tg*^ and control littermate wt mice (upper) and in the ileum (left) and colon (right) of mice with (Reg3〈/ad) or without (NC/ad) Reg3a/adenovirus injection (lower). **f**, **g** Staining of ileum mucin (**f**) and colon tissues (**g**) in human *REG3A*^*tg*^ or control littermate wt mice using IL-22-neutralizing antibody or control isotype antibody (Iso) (ten slides/mouse in **g**); scale bars = 40 µm. Student’s *t* test, mean ± SD in **a**–**e**, mean ± SEM in **g**, *n* = 6; NS no significance; RErelative expression; data are representative of three independent experiments. Also see Supplementary Figs. 3, 4

Gut immune cells may influence mucosal cell proliferation through the direct cell–cell contact or the production of cytokines, such as IL-22^9,18^. We thus assessed IL-22-associated gut immune cell population and subpopulations according to the described gated strategy (Supplementary Fig. 3). IL-22(+) cells include innate lymphocyte cell 3 (ILC3), CD4(+)Th17, and CD4(+)Th22 cells in gut tissues^19^. We found that the increased IL-22(+) cells in human *REG3A*^*tg*^ mice mainly belonged to CD4(–) IL-22(+) cells but not CD4(+)IL17(+) (Fig. 2a), implying that these cells may be ILC3 cells. ILC3 cells are RORγt-positive cells and constitute at least two bona fide subsets NCR(+) ILC3 expressing NKp46 and LTi-like ILC3, which includes CD4(+) and CD4(–) subsets^20,21^. The increased ILC3 cells in the ileum and colon of human *REG3A*^*tg*^ mice were CD45(+)lin(–)RORγt(+) IL-22(+)NKp46(–) CD4(–) ILC3 cells (Fig. 2b, c), which may strongly produce IL-22^22^. Increased CD45(+)lin(–)RORγt(+) IL-22(+)NKp46(–) CD4(–) ILC3 cells were also found in Reg3α adenovirus-injected mice (Fig. 2d and Supplementary Fig. 1b, d, f). Higher levels of IL-22 in the ileum and colon tissues were detected in *REG3A*^*tg*^ mice and Reg3α/adenovirus- injected mice (Fig. 2e). All of these imply that CD45(+)lin(–)RORγt (+) IL-22(+)NKp46(–) CD4(–) ILC3 cells may be involved in REG3A/Reg3α-mediated formation of gut mucus layers. To further confirm the role of IL-22 in REG3A-mediated gut mucus layers, we treated *REG3A*^*tg*^ mice by injecting IL-22-neutralizing antibodies. Reduced mucus gel in the ileum tissues and thinned mucus layers in the colon tissues of *REG3A*^*tg*^ mice were observed after administering IL-22-neutralizing antibodies (Fig. 2f, g), indicating that REG3A-mediated mucus layers were dependent on IL-22. In addition, the proportion of CD11C(+)CD103(+)CD11B(+) dendritic cells, CD11B(+)Ly6C(+) myeloid-derived monocytes, and CD11B(+)F4/80(+) macrophages, which may be promoted by GM-CSF from RORγt(+)IL-22(+) ILC3 cells, also increased (Supplementary Fig. 4). Taken together, gut REG3A (Reg3α in mouse) promotes the formation of gut mucus layers in the small intestine and colon through RORγt (+) IL-22(+) ILC3 cells.

### *Lactobacilli* promote the accumulation of ILC3

**Fig. 3:**
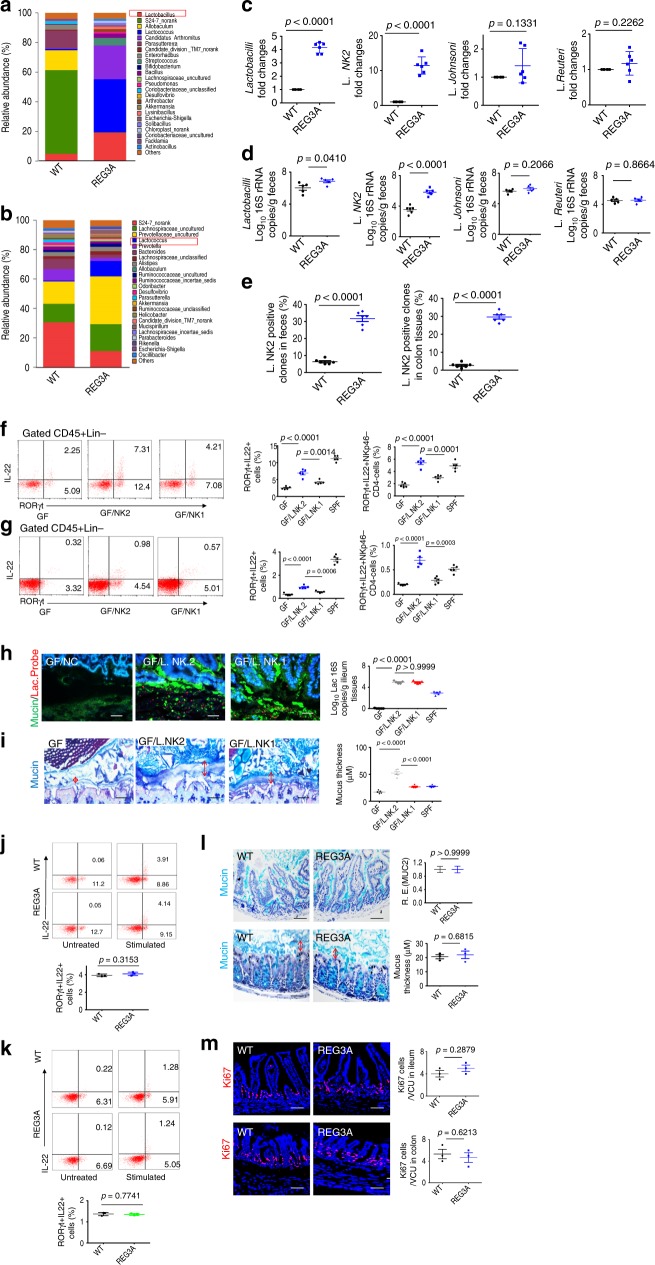
REG3A-associated *lactobacillus* is critical in increased RORγt (+)IL-22 (+) ILC3 cells in gut tissues. **a**, **b** 16S rRNA analyses of gut microbiota in the ileum contents (**a**) and colon contents (**b**) of wt and human *REG3A*^*tg*^ mice (REG3A) under normal chow (N. Chow) (*n* = 6). **c** Proportion of *lactobacillus* genus and species in the ileum of *wt* and *huREG3* 〈^*tg*^ mice (*n* = 6). **d** QPCR of *lactobacillus* genus, *lactobacillus NK2* (L.NK2), *lactobacillus Reuteri* (L. Reuteri), *and lactobacillus Johnsoni* (L. Johnsoni) in the feces**. e** Percentage of *lactobacillus NK2* (L.NK2) in the total lactobacilli of feces (left) and colon tissues (right) of wt and *human REG3A*^*tg*^ mice (*n* = 6). *Lactobacilli* in feces and colon tissues were cultured and sequenced using 16S rDNA. **f**, **g**, Flow cytometry of IL-22(+)RORγt (+) and IL-22(+)RORγt (+) NK46(–)CD4(–) cells in the ileum (**f**) and colon (**g**) LP of GF mice with or without *L. NK1* (NK1) or *L.NK2* (NK2) colonization (*n* = 6). **h** Staining of mucus layers in the ileum from GF mice with or without *L. NK2* or *L. NK1* colonization. Tissue sections were stained with anti-mucin 2 antibody (green) and hybridized to a probe that recognized 16S rRNA of *lactobacillus* (red) and a nonspecific scrambled probe and counterstained with DAPI to visualize the nuclei (blue). Ten slides/mouse were analyzed (*n* = 5). **i** Staining of mucus layers in the colon from GF mice with or without *L. NK2* or *L. NK1* colonization. Ten slides/mouse were analyzed (*n* = 5). GF mice were colonized with *L. NK2* or *L. NK1* (1⋅10^9^/mouse). **j**, **k**, Flow cytometry of ROR^©^t(+)IL22(+) cells and QRT-PCR of IL-22 in the ileum (**j**) and colon (**k**) LP of wt and *REG3A*^*tg*^ germ-free mice (*n* = 3). **l** Staining and qRT-PCR of ileum mucin and colon tissues in *REG3A*^*tg*^ and wt germ-free mice. **m** ki67 cells in the ileum and colon epithelial cells of *REG3A*^*tg*^ and control wt germ-free mice. SPF, *wt* mice raised in specific pathogen-free (SPF) environment. Student’s *t* test, mean ± SD in **c**, **e**, **l**, and **m**; ANOVA plus post-Bonferroni analysis in **f**–**i**; NS no significance; R.E relative expression. Data in **c**–**m** are representative of at least three independent experiments. Also see Supplementary Fig. 5 and Fig. 6

We next investigated whether the accumulation of CD45(+)lin(–)RORγt (+) IL-22(+)NKp46(–) CD4(–) ILC3 cells in human *REG3A*^*tg*^ mice is dependent on the altered microbiota. We performed the transplantation experiment of REG3A-shaped microbiota in pan-antibiotic-treated WT mice. More RORγt(+)IL-22(+) cells increased mucus gel in the ileum, thickened mucus layers in the colon tissues, and increased goblet cells and Ki67 cells in the gut tissues were observed in *REG3A*^tg^ feces- transplanted mice (Supplementary Fig. 5). We next analyzed the composition of gut microbiota and found that the proportion of *lactobacilli* was high in the ileum and colon of human *REG3A*^*tg*^ mice, as compared with their control cohoused littermates (Fig. 3a–e). Although mouse Reg3 may kill some Gram-positive bacteria, Gram-positive *lactobacilli* are not sensitive to Reg3^14,23^. We further analyzed the composition of *lactobacilli* via in vitro culture and sequencing analyses, and found that increased *lactobacillus* in human *REG3A*^*tg*^ mice was close to *L. murinus* isolates, which was named as *L. NK2* (Fig. 3c, d and Supplementary Fig. 6a, b). We next employed germ-free (GF) mice to examine the effects of *L. NK2* strain on RORγt (+) IL-22(+) ILC3 cells and formation of gut mucus layers (Supplementary Fig. 6c). Infusion of *L. NK2* caused increased mucus and accumulation of RORγt (+) IL-22(+) ILC3 cells (Fig. 3f, g). Non-transplanted control GF mice housed under separated but similar conditions had less RORγt(+)IL-22(+) cells (Fig. 3f, g), consistent with previous data in GF mice^24,25^. Moreover, the effect of *lactobacillus* is bacteria-species specific. *L. NK2* strain than *L. NK1* strain, which is homologous with *L. taiwaness* isolate^23^, caused more remarkable accumulation of RORγt(+)IL-22(+) cells in GF mice (Fig. 3f, g). Increased mucus gel in the ileum, thickened mucus layers in the colon tissues, and increased goblet cells and Ki67 cells in the gut tissues were also observed in *L. NK2* strain- transplanted GF mice (Fig. 3h, i and Supplementary Fig. 6d–f). The increased RORγt (+) IL-22(+) cells, thickened gut mucus, and increased Ki67 cells were also not found in germ-free REG3A tg mice (Fig. 3j–m). Thus, we demonstrate that *lactobacillus L. NK2* alone promotes the accumulation of RORγt (+) IL-22(+) in gut tissues.

### Increased ILC3 is related to *lactobacillus*-induced L-Kyn

**Fig. 4:**
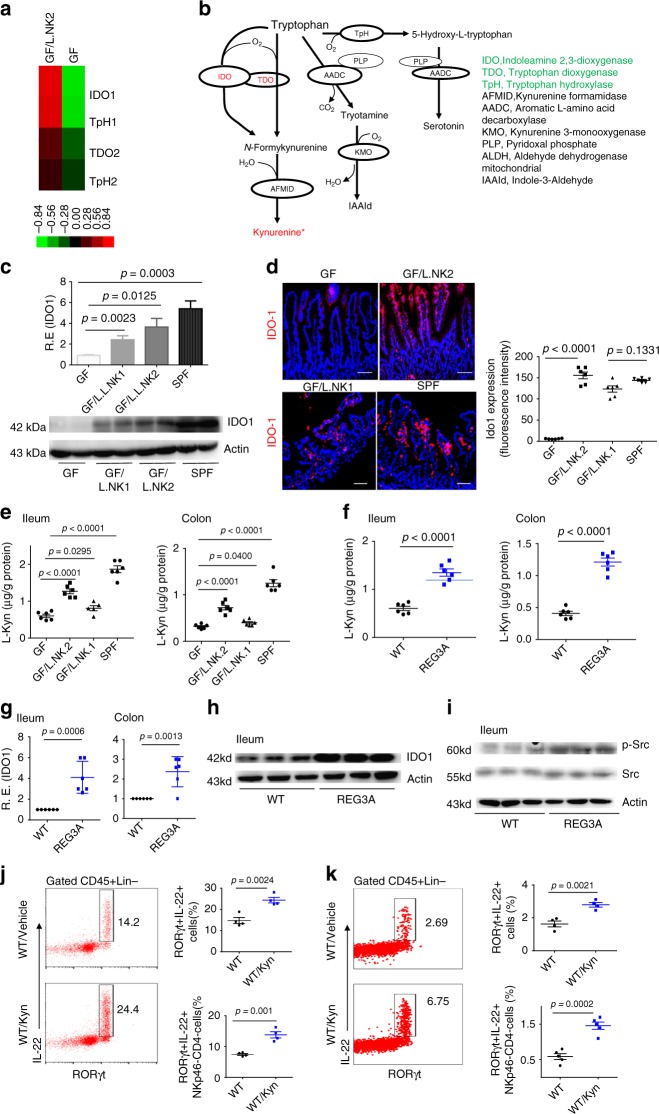
REG3A-associated *lactobacillus* promotes production of L-Kyn in gut epithelial cells. **a** Microarray of the ileum epithelial cells in REG3A-associated *lactobacillus-*colonized GF mice (GF/L.NK2) and control uncolonized GF mice (*n* = 6). **b** Metabolism map of tryptophan in mouse gut epithelial cells. **c** QRT-PCR and immunoblotting of IDO1 in the ileum tissues of L. NK2 or L. NK1-colonized GF mice and control uncolonized GF mice. SPF, wt mice raised in SPF environment. **d** Immunostaining of IDO1 in the ileum tissues of REG3A-associated *lactobacillus-*colonized GF mice and control GF mice (representative image, *n* = 6). **e** L-Kyn ELISA of the ileum (left) and colon (right) epithelial cells of REG3A-associated *lactobacillus-*colonized GF mice and control GF mice (*n* = 6). **f** L-Kyn ELISA of the ileum (left) and colon (right) epithelial cells of human *REG3A* ^*tg*^ mice and their control littermates (*n* = 6). **g**, **h** QRT-PCR (**g**) and immunoblotting (**h**) of IDO1 in the ileum or colon epithelial cells of human *REG3A*^*tg*^ mice and their control littermates. **i** Immunostaining of p-Src in the ileum epithelial cells of human *REG3A*^*tg*^ mice and their control littermates. **j**, **k** Flow cytometry of CD45(+)RORγt(+)IL-22(+) and their subsets in the ileum (**j**) and colon (**k**) LP of mice with or without L-Kyn infusion. Scale bars = 40 µm; Student’s *t* test was used in **f**, **g**, **j**, and **k**. ANOVA plus post-Bonferroni analysis in **c**, **d**, and **e**; NS no significance; RE relative expression. In **h** and **i**, different individuals were indicated

We next determined how REG3A-associated *lactobacillus* was formed to cause the accumulation of CD45(+)Lin(–)ROR□t(+)IL-22(+) cells. Previous studies showed that AhR ligand indole-3-aldehyde (IAld) from *lactobacillus* may contribute to AhR-dependent IL-22 transcription^10^. However, this was not the case for REG3A-associated *lactobacillus* (Supplementary Fig. 7). Studies have also shown that diverse host-derived signals can regulate and cause the accumulation of ROR^©^t(+)IL-22(+) cells, such as AhR ligands derived/generated from host cells^26,27^, chemotaxis, and IL-23 by CX_3_CR1(+) mononuclear phagocytes^28^. To investigate the factor(s) which is responsible for an increase in CD45(+)Lin(–)ROR^©^t(+)IL-22(+) cells, we employed a microarray to compare the gene expression of gut ileum epithelial cells and gut immune tissues (Payer’s patch node). We did not find ROR^©^t(+)IL-22(+) cells associated with chemokines and/or IL-23 (GSE111111). Interestingly, *L. NK2* colonization in GF mice induced at least a twofold change in the expression of multiple other genes in gut epithelial cells, typically indoleamine 2,3-dioxygenase 1 (IDO1) (Fig. 4a and GSE111111), which is a critical enzyme for tryptophan (Trp) metabolism to produce AhR ligends such as L-Kyn (Fig. 4b)^26,27^. QRT-PCR and immunoblotting also exhibited the higher expression of IDO1 in the gut epithelial tissues of lactobacillus-infused mice (Fig. 4c and Supplementary Fig. 11). Importantly, IDO1 was mainly expressed in gut epithelial cells in *L. NK2*-infused mice (Fig. 4d). Since Trp metabolites by IDO1 are primarily L-Kyn, more L-Kyn was detected in the gut epithelial cells of *L. NK2*-infused GF mice than control *lactobacillus* (Fig. 4e). L-Kyn in the gut epithelial cells of *REG3A*^*tg*^ mice was higher (Fig. 4f). Trp metabolism-associated components such as IDO1 and p-Src^29^ were also higher in *REG3A*^*tg*^ mice (Fig. 4g–i and Supplementary Fig. 11). In vivo-administered L-Kyn caused the accumulation of ROR^©^t (+)IL-22(+) cells in both the ileum and colon tissues (Fig. 4j, k). Thus, the AhR ligand L-Kyn produced by gut epithelial cells is responsible for *lactobacillus*-mediated ROR γt(+)IL-22(+) cells.

### *Lactobacillus*-derived L-Orn is a critical factor for ILC3

**Fig. 5:**
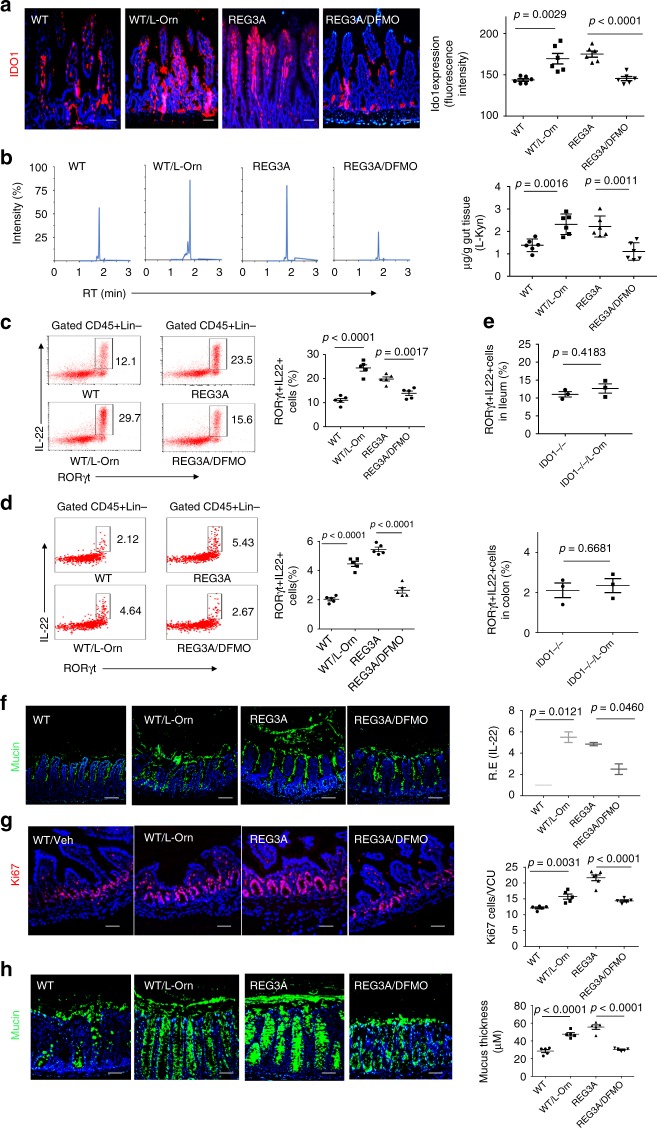
*Lactobacillus-*derived L-Orn promotes the production of AhR ligand L-Kyn in gut epithelial cells. **a** Immunostaining of IDO1 in the ileum after L-Orn administration in wt mice or L-Orn inhibitor DFMO administration in human *REG3A*^*tg*^ mice. **b** HPLC/MASS of L-Kyn in the ileum epithelial cells after administrating L-Orn or L-Orn inhibitor DFMO (*n* = 6). **c**, **d** Flow cytometry of RORγt (+) IL-22(+) cells in the ileum (**c**) and colon (**d**) of mice after administering L-Orn or L-Orn inhibitor DFMO. **e** Flow cytometry of RORγt (+) IL-22(+) cells in the ileum and colon of IDO-1 KO with or withour L-Orn. **f** Immunostaining and qRT-PCR of mucin in the ileum of mice after administering L-Orn or L-Orn inhibitor DFMO (ten slides/mouse, *n* = 6). **g** Staining of Ki67 cells in the ileum of mice after administering L-Orn or L-Orn inhibitor DFMO (ten slides/mouse, *n* = 6). **h** Immunostaining of mucin in the colon of mice after administering L-Orn or L-Orn inhibitor DFMO (ten slides/mouse, *n* = 6). WT, wild-type mice; WT/Orn, L-Orn-fed mice; REG3A, human *REG3A*^*tg*^ mice; REG3A/DFMO, L-Orn inhibitor DFMO-fed mice. IDO-1 KO/L-Orn, L-Orn-fed IDO-1 KO mice. Scale bars = 40 µm; Student’s *t* test, mean ± SD in e; ANOVA plus post-Bonferroni analysis in **a**, **b**, **c**, **d**, **f**, **g**, and **h**; NS no significance; RE, relative expression. Data are representative of at least three independent experiments. Also see Supplementary Fig. 7

We next sought to address how *lactobacillus* regulates the expression of IDO1 in gut epithelial cells. In addition to cytokine-mediated activation, IDO1 signaling can also be triggered by metabolites such as L-Orn^29^; thus, we hypothesized that REG3A-associated *lactobacillus* might produce some metabolites to regulate the expression and activity of IDO1. Indeed, there was increased L-Orn in the gut contents of GF mice with *L. NK2* colonization (Supplementary Fig. 8a). Increased L-Orn was further confirmed by ELISA in the ileum but also in the colon contents (Supplementary Fig. 8b). Higher levels of L-Orn were also detected in the contents of the ileum and colon of *REG3A*^*tg*^ mice (Supplementary Fig. 8c). L-Orn may upregulate IDO1 in macrophages and dendritic cells^30^. When gut ileum and colon epithelial cells were exposed to different concentrations of L-Orn in vitro, L-Orn also upregulated the expression of IDO1 in these tissues (Supplementary Fig. 8d, e). Importantly, L-Orn-infused mice had a high level of IDO1 in their gut epithelial cells; whereas L-Orn inhibitor DFMO, which may inhibit L-Orn to putrescine^29^, suppressed the expression of IDO1 (Supplementary Fig. 8f, g and Fig. 5a). Notably, spermidine, a metabolite of L-Orn also induced the expression of IDO-1 (Supplementary Fig. 8h), implying that L-Orn-mediated IDO-1 expression may be through its metabolites. L-Kyn increased in the epithelial cells in L-Orn-infused mice, but DFMO caused reduced L-Kyn in *REG3A*^*tg*^ mice (Fig. 5b). Thus, lactobacillus-derived L-Orn promotes the production of the AhR ligand L-Kyn in gut epithelial cells. Increased RORγt (+) IL-22(+) ILC3 cell populations and higher levels of IL-22 were also detected in the ileum and colon of L-Orn-infused mice (Fig. 5c, d). Conversely, L-Orn inhibitor (DFMO) decreased accumulation of RORγt (+) IL-22(+) cells in the gut tissues (Fig. 5c, d). Unsimilar to wt mice, IDO-1 KO mice did not exhibit the same responses to L-Orn (Fig. 5e). Administration of L-Orn also promoted mucin secretion, goblet cell production, and cell proliferation in wt mice. Conversely, L-Orn inhibitor-infused *REG3A*^*tg*^ mice had reduced mucin secretion, goblet cell production, and cell proliferation (Fig. 5f–h). Thus, *lactobacillus*-derived L-Orn is a critical factor for *lactobacillus*-mediated ROR^©^t(+)IL-22(+) cells and gut mucus formation.

### L-OCT deficiency impedes the effect of *lactobacillus*

L-Orn may be derived from Arg metabolism through the ADI pathway in *lactobacillus* (Supplementary Fig. 9a)^31^. Indeed, *L. NK2* produced L-Orn through Arg metabolism, but *L. NK2* and *L. reuteri* more effectively used Arg to produce L-Orn than *L. NK1* (Supplementary Fig. 9b). The ADI pathway comprises three reactions catalyzed by Arg deminase (ADI; EC3.5.3.6), ornithine carbamoyl-transferase (OCT: EC2.1.3.3), and carbamate kinase (CK: EC 2.7.2.2), leading to the conversion of Arg into ornithine (Supplementary Fig. 9a)^31^. We prepared *L. reuteri*ΔOCT but failed to generate *L. NK2*ΔOCT. The generated *L. reuteri*ΔOCT did not produce L-Orn in vitro in the presence of Arg (Supplementary Fig. 9b). OCT deficiency affected the concentration of L-Kyn not only in the ileum but also in the colon epithelial tissues (Supplementary Fig. 9c). The levels of both IDO1 and p-Src in the gut epithelial cells of OCT-deficient *lactobacillus*-colonized mice were lower (Supplementary Fig. 9d–f). The proportion of CD45(+) RORγt(+)IL-22(+)lin(–)NKp46(–)CD4(–) ILC3 cells, decreased mucus gel, thinner mucus layers, and reduced mucin 2 were also observed in these OCT-deficient *lactobacillus*-colonized mice (Supplementary Fig. 9g–i). *L. reuteri* also produced the AhR ligand indole-3-aldehyde^10^. But no differences were detected in L-Kyn in the gut content between wild-type *L. reuteri* and OCT-deficient *L. reuteri*. Higher levels of L-Orn could be detected in *L. reuteri* and *L.NK2*-infused mice (Supplementary Fig. 9j). Both *L. reuteri* and OCT- deficient *L. reuteri* had a similar proliferative ability (Supplementary Fig. 9k).

**Fig. 6:**
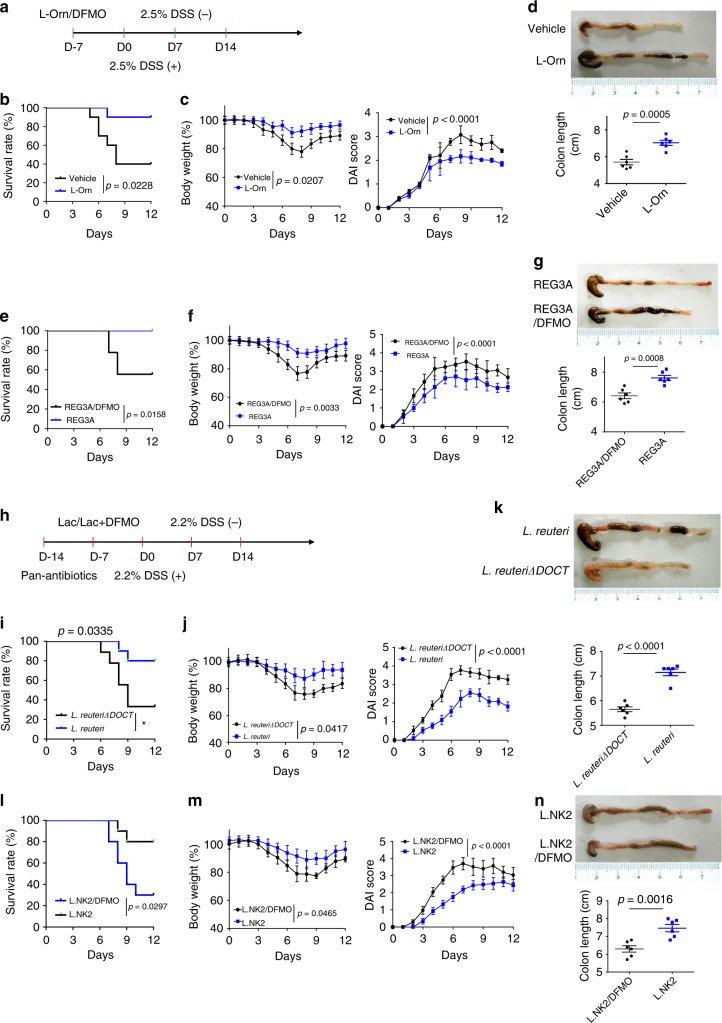
*Lactobacillus-*derived L-Orn promotes resistance to DSS-mediated colitis. **a** Experimental design for B–G. **b**, **c**, Survival rate (**b**) and body weight and the disease activity score (**c**) were monitored after the start of DSS. Wild-type (WT) mice with (L-Orn) or without (Vehicle) administration of L-Orn (*n* = 18, male). **d** Length of colon tissue in wild-type mice with or without administration of L-Orn. Mice were killed on day 7 after the start of DSS and colon length was measured. **e**, **f**, Survival rate (**e**) and body weight and the disease activity index (**f**) were monitored after the start of DSS. Human *REG3A*^*tg*^ *mice* with (REG3a/DFMO) or without (REG3A/Vehicle) administration of DFMO (*n* = 18, male) mice. **g** Length of colon tissue in human *REG3A*^*tg*^ *mice* with or without administration of DFMO mice. Mice were killed on day 7 after the start of DSS and colon length was measured. **h** Experimental design for **i**–**n**. **i**, **j**, Survival rate (**i**) and body weight and the disease activity score (**j**) were monitored after the start of DSS. Mice were infused with *L. reuteri* or *L. reuteri/ΔOCT* (*n* = 18, male). **k** Length of colon tissue in mice after infusing *L. reuteri* or *L. reuteri/ΔOCT*. Mice were killed on day 7 after the start of DSS and colon length was measured. **l**, **m**, Survival rate (**l**) and body weight and the disease activity score (**m**) were monitored after the start of DSS. Mice were infused with *or L. NK2 with DFMO* (*n* = 18, male) mice. **n** Length of colon tissue in mice after infusing *L. NK2 or L. NK2* with DFMO. Mice were killed on day 7 after the start of DSS and colon length was measured. Wilcoxon’s test in **b**, **e**, **i**, and **l**; analysis of variance test in **c**, **f**, **j**, and **m**; Student’s *t* test in **d**, **g**, **k**, and **n**; NS no significance; RE relative expression. Data are representative of three independent experiments. Also see Supplementary Fig. 9

Since *L.NK2* promotes the homeostasis of not only the small intestine but also the colon, we employed DSS- mediated colitic model to assess the physiological function of *lactobacillus*-derived L-Orn in resisting DSS- mediated colitis. L-Orn administration promoted resistance of wt mice to DSS-induced colitis; whereas L-Orn inhibitor decreased the resistance of human *REG3A*^*tg*^ to DSS-mediated colitis (Fig. 6a–g and Supplementary Fig. 10a, b, e, f). The levels of serum LPS were lower and the numbers of bacterium clones in the spleen were less in L-Orn-administered mice (Supplementary Fig. 10c, d); whereas there were higher levels of serum LPS and the more bacterium clones in L-Orn inhibitor administered human *REG3A*^*tg*^ mice after giving DSS (Supplementary Fig. 10g, h). OCT-deficient *lactobacillus*, which did not produce L-Orn, reduced the resistance of mice to DSS-mediated colitis (Fig. 6i–k and Supplementary Fig. 10i, j). Similar effectiveness was also observed in *L. NK2-*transplanted mice, which were administered using L-Orn inhibitor DFMO (Fig. 6l–n and Supplementary Fig. 10m, n). They also had higher levels of serum LPS and the more bacteria in the spleen of OCT-deficient *lactobacillus*-infused mice and also in L. NK2 transplantation with L-Orn inhibitor- treated mice after giving DSS (Supplementary Fig. 10k, l, o, p). These results demonstrate that *lactobacillus*- derived L-Orn plays a critical role in maintaining gut mucosal homeostasis.

## Discussion

We found that *lactobacillus* may promote the homeostasis of gut mucus layer through producing L-Orn. L-Orn stimulates Trp metabolism to produce AhR ligands in gut epithelial cells, which induce accumulation of RORγt (+) IL-22(+) ILC3 in gut tissues. We demonstrate that the proportion of L-Orn-producing *lactobacilli* in the gut contents may be regulated by gut epithelial REG3A. Thus, there exists a gut epithelial REG3A—*lactobacillus*-derived L-Orn—L-Kyn in gut epithelial cells—RORγt (+) IL-22(+) ILC3 immune cell axis to maintain gut mucosal homeostasis. Our data improve understanding of the mechanism of gut mucosal homeostasis. Since the gut mucosal homeostasis plays a critical role in human diseases such as colitis and metabolism-associated diseases, our findings also offer insight for prevention and treatment of these diseases.

We demonstrate that *lactobacillus*-derived L-Orn may upregulate IDO1 in gut epithelial cells to produce AhR ligand L-Kyn. AhR ligands that drive the differentiation of ILC3 immune cells may be entirely derived from the endogenous ligands, such as the Trp metabolites L-Kyn^24,27^. Several cell types, including specific subsets of dendritic cells (DCs), macrophages, and immature monocytes, express increased levels of IDO1, which may promote the AhR ligand production in response to inflammatory cues, such as interferon γ (IFNγ) or signal transducer and activator of transcription 3 (STAT3)-activity stimuli^32^, and CpG oligodeoxynucleotides (ODNs)^32^. However, our data exhibit that gut epithelial cells also produce AhR ligand L-Kyn by L-Orn through upregulating IDO1. Previous studies show that IDO1-positive staining may be primarily detected within the interstitial space of the villi or mucosal layer^33^. IDO1 expression at mucosal sites may be modulated during immune activation^33^. Recent studies also reveal that L-Orn may upregulate IDO1 in the macrophages and DCs^29^.

*Lactobacillus* may produce L-Orn through arginine (Arg) metabolisms. Others also reported that *lactobacillus* could produce L-Orn^30,34^. Interestingly, Arg has been found to preserve intestinal barrier integrity in animals after intestinal obstruction^35^ and dextran sodium sulfate (DSS) colitis. It has also been established that Arg may improve the migration of epithelial cells, increase villus height and crypt depth, and decrease cell apoptosis in methotrexate (MTX)-induced mucositis and DSS colitis^35^. Long-term increased polyamine (L-Orn metabolites) intake elevated blood spermine levels and inhibited aging-associated pathologies in mice and humans^36^. Since L-Orn from *lactobacillus* through Arg metabolism may promote gut mucus homeostasis, our results expand understanding of the mechanism by which Arg contributes to this homeostasis. Others also found that some lactobacilli, such as *L. reuteri*, have an effect on IL-22 production by producing indole-3-aldehyde^10^. Thus, multiple *lactobacillus* strains or multiple effects of one lactobacillus strain are involved in the regulation of RORγt (+)IL-22(+) ILC3 cells through different mechanisms. However, previous reports showed that REG3α could produce cytotoxity to Gram + bacteria. This may be that Lactobacillus is different from other Gram+ bacteria. It is possible that the cytotoxity of REG3 to other Gram+ bacteria may affect proliferation and growth of *lactobacilli*. There also exist increased Gram-positive *lactobacilli* in other *Reg*^*tg*^ *mice*^14,23^.

## Methods

### Mice

Four- to six-week-old male or female C57BL/6 mice were obtained from Nanjing Animal Center. IDO-1^–/–^ mice were from Nanjing Animal Center. All experimental litters were bred and maintained under specific pathogen-free conditions in the Animal Center of Nankai University. Experiments were carried out using age- and gender-matched mice. All procedures were conducted according to the Institutional Animal Care and Use Committee of the Model Animal Research Center. Animal experiments were approved by the Institute’s Animal Ethics Committee of Nankai University. All experimental variables such as husbandry, parental genotypes, and environmental influences were carefully controlled. C57BL/6 germ-free (GF) mice were generated by Shanghai SLAC Laboratory Animal Co. LID. All experiments in GF mice were performed in Shanghai SLAC Laboratory Animal Co. LID. Human REG3A transgenic mice (human *REG3A*^*tg*^) mice were prepared by Nanjing Animal Center. HD5 promoter, which may specifically promote the REG3A expression in gut Paneth cells, was conjugated into Pinsulator–pHD5-promoter–CDS–polyplasmid. The fragments of REG3A–CDS and polyA were cloned into HD5 promoter–Pinsulator. This conjugation was demonstrated using primers (M13F: GCCAGGGTTTTCCCAGTCACGA and HD5-REG3A-tR: GTAGGGTATGATGTGACGTTTG) and sequencing using the primers (HD5-tR:CAGCATGGTGGTACATGCCT and CDS-tF: GGCAACATATGCCCATATGC). Human *REG3A*^*tg*^ mice were identified using the following primers (REG3A-tF3: GAGCCCAATGGAGAAGGTTGG and REG3A -tR3:GTCCTTCCGAGTGAGAGACAC, which produced a 323-bp band in tg mice and no band in wt mice). According to this method, mice were identified as human REG3A-positive mice (human *REG3A*^*tg*^) and human REG3A-negative mice (wt). Human *REG3A*^*tg*^ mice or wt siblings were from a cross between wt mice and human *REG3A*^*tg*^ mice (heterozygous mice). The mice were from different mothers.

For preparation of mouse Reg3α/adenovirus-injected mice, mouse Reg3α adenoviruses (Reg3α/Ad) were first prepared by ABM, Canada and expanded by JIKAI, China, and then ip injected into mice according to the indicated time (1 × 10^9^ viral particles/mouse). Control empty adenoviruses (NC/Ad, 1 × 10^9^ control viral particles) were from ABM, Canada.

### Mouse models

For DSS-induced colitis, dextran sodium sulfate (DSS)-induced colitis was performed according to the previous method^37^. Briefly, mice received 2.5% (wt/vol) DSS (40,000 kDa; ICN Biochemicals) or indicated doses in their drinking water for 7 days, and then switched to regular drinking water. The amount of DSS water drank per animal was recorded and no differences in intake between strains were observed. For survival studies, mice were followed for 14 days post start of DSS treatment. Mice were weighed every other day for the determination of percent weight change. This was calculated as % weight change = (weight at day X–day 0/weight at day 0) × 100. Diarrhea was scored daily as follows: 0, normal; 2, loose stools; 4, watery diarrhea. Blood in stool was scored as follows: 0, normal; 2, slight bleeding; 4, gross bleeding. Weight loss was scored as follows: 0, none; 1, 1–5%; 2, 5–10%; 3, 10–15%; 4, >15%. Disease activity index was the average of these scores: (combined score of stool consistency, bleeding, and weight loss)/3^38^. Mice were killed at the indicated days for histological study. Representative colon tissues were embedded in paraffin for hematoxylin/eosin (H&E) staining or embedded in OCT compound (Tissue-Tek, Sakura, Torrance, CA) and frozen over liquid nitrogen for immunostaining. For histological evaluation, colonic epithelial damage was scored blindly as follows: 0, normal; 1, hyperproliferation, irregular crypts, and goblet cell loss; 2, mild-to-moderate crypt loss (10–50%); 3, severe crypt loss (50–90%); 4, complete crypt loss, surface epithelium intact; 5, small-to-medium-sized ulcer (<10 crypt widths); 6, large ulcer (>10 crypt widths)^39^.

For microbiota transplantation, germ-free mice were orally administered 200 □l of *lactobacillus* (1 × 10^9^ bacteria, once/week). In wt mice, mice were first treated with ampicillin (A, 1 g/L, Sigma), vancomycin (V, 0.5 g/L), neomycin sulfate (N, 1 g/L), and metronidazole (M, 1 g/L) via the drinking water for 2 weeks. To confirm the elimination of bacteria, stools were collected from antibiotic-treated and untreated mice and cultured in anaerobic and aerobic conditions. Mice were orally administered 200 □l of fecal suspension or 1 × 10^9^ bacteria (once/week).

For L-kynurenine (L-Kyn) administration, mice were randomly assigned to two different treatment groups (*n* = 6/group). L-kynurenine sulfate (300 mg/kg, i.p.) or vehicle (0.1 M PBS buffer) of the same volume (0.2 ml) was administered intraperitoneally^40^.

For L-Orn or eflornithine (DFMO) infusion, mice were randomly assigned to two different treatment groups (*n* = 6/group), and then mice were administered in drinking distilled H_2_O for 14 days. The mean L-Orn consumption of mice was ∼3.3 g/kg/d^41^. Eflornithine (DFMO) (MedChem Express) was administered as a 1% solution in drinking distilled H_2_O to mice for 14 days^42^. The mean DFMO consumption of mice was ∼1.5 g/kg/d. Mice fed with H_2_O without L-Orn or DFMO were used as control.

### Gut microbiota analysis

Gut microbiota was analyzed according to our previously reported method^23^. Briefly, gut microbiota were analyzed by Majorbio Biotechnology Company (Shanghai, China) using primers that target to the V3–V4 regions of 16S rRNA. Operational Taxonomic Unit (OTU) analysis was performed as follows: sequences were processed (trimmed) using the Mothur software and subsequently clustered at 97% sequence identity using cd-hit to generate OTUs. The OTU memberships of the sequences were used to construct a sample–OTU count matrix. The samples were clustered at genus and OTU levels using the sample–genus and sample–OTU count matrices, respectively. For each clustering, Morisita–Horn dissimilarity was used to compute a sample distance matrix from the initial count matrix, and the distance matrix was subsequently used to generate a hierarchical clustering using Ward’s minimum variance method. For the absolute numbers of gut *lactobacilli*, 16Ss rRNAs were extracted, and then amplified using strain-specific primers. The concentration of each product was detected and then exchanged into copy numbers. Standard curves were prepared from serial dilution of *Lactobacillus* genomic 16S rRNAs. Primers used were listed in Supplementary Table 1.

### *Lactobacillus* isolation and culture

*Lactobacillus* isolation and culture were performed according to previous method^23^. In brief, 100 mg of fresh fecal samples were collected and diluted in 2 ml of BPS solution, and cultured on Rogosa SL selective medium (Sigma-Aldrich) for *lactobacillus* enumeration, and then colonies were identified and purified using 16S rRNA sequence analyses. *Lactobacilli* were cultured in deMan, Rogosa, Sharpe (MRS; 3 M Health Care, St. Paul, MN) media and also grown on MRS agar containing 10% sucrose. Anaerobic conditions were generated with the sachets of AnaeroPack-Anaero (Mitsubishi Gas Chemical, Japan) in an airtight jar. After 24 h of cultivation in liquid media, *lactobacilli* could reach 1 × 10^9^ CFU/ml.

For L-Orn production by *lactobacillus* in vitro, *lactobacilli* were propagated routinely for 24 h at 37 °C in MRS broth medium. Before using to assay arginine catabolism, cells were first subcultured (37 °C for 24 h) on MRS agar. Monoclonal *lactobacillus* was newly propagated in MRS broth with or without 6 mM arginine, which was then used to induce arginine catabolism. The supernatants were collected at the indicated time and L-ornithine was analyzed using ELISA.

### Construction of OCT-deficient *lactobacillus*

Ornithine carbamoyltransferase (OCT) nucleotide sequences of *L. reuteri* DSM 20016 (NCBI GI 148530277, Lreu_0044) and JCM 1112 (NCBI GI 183223999, LAR_0041) were used for identifying a homologous gene in the genome of *L. reuteri* ATCC PTA 4659. The locus of the gene encoding the OCT and flanking nucleotide sequences in *L. reuteri* ATCC PTA 4659 were analyzed with the BLAST program against the NCBI databases (http://blast.ncbi.nlm.nih.gov/Blast. cgi). The gene coding for OCT in *L. reuteri* ATCC PTA 4659 was truncated according to the following method. To construct a ΔOCT (ornithine carbamoyltransferase) deletion mutant inserted with chloramphenicol acetyltransferase (*cat*, Cm), the upstream chromosomal DNA fragment (896 bp) and the downstream chromosomal DNA fragment (949 bp) and the *cat* gene were amplified with the Left-F/Left-R, Right-F/Right-R, and Cat-F/Cat-R primers. The vector pMG36e fragment was amplified by 36e-F/36e-R primers. Then the four PCR products were gel purified, ligated by the one-step cloning kit, and transformed into *E. coli* DH5α. The recombinant plasmid pMG36e-left-cat-right was electroporated into *L. reuteri* with selection on MRS medium of 3 μg/ml erythromycin (Em) and 3 μg/ml Cm. The recombinant *L. reuteri*-containing plasmid pMG36e-left-cat-right was propagated in MRS medium with 5 μg/ml Em at 37 ℃ for 30 generations without Em at 37 ℃ for 20 generations for the loss of plasmid subsequently. Then the cultures of the 20th generation were serially diluted from 10 to 10^7^ in sterilized PBS and plated onto MRS medium without any antibiotics. After culturing for 48 h, the clones were spotted on the MRS medium with Em and Cm. After incubation at 37 °C for 48 h, the clones which were growing only on the MRS medium with Cm, were thought to be desired mutants. The sequences around the OCT gene were amplified and sequenced to confirm the mutants. PCR primers used in this study were listed in Supplementary Table 1.

### Microarray

Expression of coding mRNA was analyzed by Beijing Capitalbio Technology Co., Ltd, according to our previously reported method^43^. Total RNA was extracted using Trizol (Life Technologies). Contaminating DNAs were removed using RNeasy spin columns (Qiagen). The quality of isolated RNA samples was evaluated with an Agilent Bioanalyzer 2100 (Agilent Technologies) and the purified RNA was quantified using a NanoDrop ND-2000 spectrophotometer (Thermo Fisher). The Agilent Gene Expression oligo microarrays and miRNA microarrays were analyzed using Agilent Gene Expression oligo microarrays Version 6.5, May 2010 and Agilent miRNA microarrays Version 2.3. The R software (v.2.13.0) platform was applied to analyze the microarray data, and the LIMMA (linear regression model) package was used to statistically analyze differentially expressed genes. Genes having a fold change >2 or <−2 and an adjusted *p* < 0.05 were considered as differentially expressed.

### Flow-cytometry analyses

Single-cell suspensions of Peyer’s patches (PP) and spleen of mice were prepared by mashing in a cell strainer (70 mm), stained, and analyzed by flow cytometry according to previous method^44^. In brief, colon or small intestine were isolated and cleaned by shaking in ice-cold PBS four times before tissue was cut into 1 -cm pieces. The epithelial cells were removed by incubating the tissue in HBSS with 2 mM EDTA for 30 min at 37 ℃ with shaking. The LP cells were isolated by incubating the tissues in digestion buffer (DMEM, 5% fetal bovine serum, and 1 mg/ml Collagenase IV and DNase I) for 40 min. The digested tissues were then filtered through a 40-mm filter. Cells were resuspended in 10 ml of the 40% fraction of a 40:80 Percoll gradient and overlaid on 5 ml of the 80% fraction in a 15-ml Falcon tube. LP cells were collected at the interphase of the Percoll gradient, washed, and resuspended in a medium, and then stained and analyzed by flow cytometry. Dead cells were eliminated through 7-AAD staining.

For intracellular staining, the cells were cultured and stimulated for 6 h with 50 ng/ml phorbol 12-myristate 13-acetate and 1 μg/ml ionomycin (Sigma) in the presence of GolgiStop. After incubation for 6 h, cells were washed in PBS, and then fixed in Cytofix/Cytoperm, permeabilized with Perm/Wash buffer, and stained with FITC-, PE-, APC- APC/cy7-, PerCP/Cy5.5-, or PE/cy7-conjugated antibodies. Meanwhile, dead cells were eliminated through 7-AAD staining.

For intracellular IL-22 staining, cells were stimulated directly ex vivo by incubating for 6 h with 20 ng/ml rIL-23 in the presence of GolgiStop for the final 3 h of culture. Cells were fixed and permeabilized by using perm buffer set, as described by the manufacturers, and stained with IL-22 and RORγt antibodies.

### Histological and immunostaining

For hematoxylin/eosin (H&E) staining, previously reported methods were used in this experiment^37^. Immunostaining was performed according to our previous method^37,45^. For RORγt(+)IL-22(+) cell staining, slides were treated with 0.1% Triton X-100, blocked with 3% H_2_O_2_. Then sections were blocked with 5% rabbit serum. Add 1:200 RORγt and IL-22 antibodies in Perm buffer for incubation overnight. Sections were then incubated with biotin-labeled goat anti-rat secondary antibody. Tyramide signal amplification was performed to RORγt staining. Fluorescence intensity was analyzed using ImageJ software.

Alcian blue-periodic acid Schiff staining was used for mucin or goblet cells.

### Immunostaining of mucus layers and localization of bacteria by fluorescent in situ hybridization

Mucus immune staining was paired with fluorescent in situ hybridization (FISH) in order to analyze bacteria localization at the surface of the intestinal mucosa according to a reported method^23^. In brief, 5-μm sections were cut and dewaxed by preheating at 60 °C for 10 min, followed by bathing in xylene at 60 °C for 10 min, xylene at room temperature for 10 min, and 99.5% ethanol for 10 min. The hybridization step was performed at 50  °C overnight with a probe (EUB338 probe (5ʹ-GCTGCCTCCCGTAGGAGT-3ʹ with a 5ʹ Cy3 label for all bacteria, Huada, China and PNA probe (Lac663) FAM-O-ACATGGAGTTCCACT for *lactobacillus*, which were synthesized by PNA BIO INC) diluted to a final concentration of 0.01 μg/mL in hybridization buffer (20 mM Tris-HCl, pH 7.4, 0.9 M NaCl, 0.1% SDS, and 20% formamide). After washing for 10 min in wash buffer (20 mM Tris-HCl, pH 7.4, 0.9 M NaCl) and 10 min in PBS, block solution (5% FBS in PBS) was added for 30 min at 50 °C. Mucin 2 primary antibody was diluted to 1:200 in block solution and applied overnight at 4  °C. After washing in PBS, block solution containing anti-rabbit secondary antibody diluted to 1:200 was applied to the section for 2 h. Nuclei were stained using Hoechst33342. Observations were performed with a Zeiss LSM 700 confocal microscope with software Zen 2011 version 7.1. This software was used to determine the distance between bacteria and the epithelial cell monolayer, as well as the mucus thickness.

### L-kynurenine HPLC–MS and HPLC–MS/MS analyses

HPLC–MS and HPLC–MS/MS analyses of L-kynurenine were performed by ProfLeader Biotechnology Company (Shanghai, China). Briefly, the UPLC–MS/MS analysis was performed on a Waters Acquity UPLC system coupled with a Waters Xevo-TQXS system. The mobile phase consisted of 20 mM ammonium acetate in water (A) and acetonitrile (B). The chromatographic separation was conducted by a gradient elution program as follows: 0 min, 5% B; 1 min, 5% B; 2 min, 25% B; 4 min, 60% B; 4.5 min, 95% B; 6 min, 95% B; 6.5 min, 5% B; 10 min, 5% B. The flow rate was 0.4 ml/min. Column temperature was 50 °C.

### HPLC/MASS analyses of gut contents

For HPLC/MASS analyses of the gut contents, 50 mg of sample were applied to the extraction procedure, and extracted with 800 μL of methanol^46^. In total, 10 μL of internal standard (2.9 mg/mL, DL-o-chlorophenylalanine) was then added. All samples were grinded to fine powder using a grinding mill at 65 Hz for 90 s. The samples after grinding were vortexed for 30 s, and centrifuged at 12,000 rpm and 4 °C for 15 min. In total, 200 μL of supernatant was transferred to a vial for HPLC–MS analysis.

### Ex vivo ileum and colon stimulation

For ex vivo ileum and colon stimulation, the fragmented fresh ileum and colon from untreated mice were immediately added in 2 ml of RPMI-1640 medium containing 10% heat-inactivated FBS (Gibco, Invitrogen), 100 U penicillin, 100 g/ml streptomycin, and 10 mm HEPES (Gibco, Invitrogen), and then L-ornithine was added into culture at the indicated concentration and time. For IDO1 analyses, the ileum or colon epithelial cells were separated from ileum or colon tissues using 0.1% EDTA, and expression of IDO1 was analyzed using qRT-PCR and immunoblotting.

### ELISA

For ELISA of L-kynurenine and L-ornithine, the preparation of tissue homogenates was performed as previously described^47,48^. L-kynurenine and L-ornithine concentration in tissue homogenates or cell culture supernatants was measured using the L-kynurenine or L-Ornithine ELISA kit (ImmuSmol).

### Western blotting

Cell lysates were denatured and subjected to SDS-PAGE, and then were transferred to PVDF membranes according to our previous methods^41,46^. Briefly, hybridizations with primary Abs were performed for 1 h at room temperature in blocking buffer. The protein–Ab complexes were checked using peroxidase-conjugated secondary Abs (Boehringer Mannheim) and ECL (Amersham Biosciences). The primary and secondary antibodies were listed in Supplementary Table 1.

### RT-PCR and qRT-PCR

RT-PCR and qRT-PCR were performed according to our previous methods^41,46^. Briefly, total RNA was extracted from cells by using TRIzol reagent (Life Technologies, Carlsbad, CA) and was transcribed to cDNA using HiFiScript cDNA Synthesis Kit (CWBIO, Beijing, China) according to the manufacturer’s instructions, and then RT-PCR was done. qRT-PCR was performed by using Hieff^TM^qPCR SYBR-Green Master Mix (YEASEN, Shanghai, China) in a Bio-Rad iQ5 multicolor RT-PCR system. The levels of each gene were calculated using the 2−ΔΔCT method. GAPDH was used as the endogenous control. The primers used for qRT-PCR were shown in Supplementary Table 1.

### Statistical analyses

Student’s *t* test, one-way analysis of variance (ANOVA), ANOVA plus post-Bonferroni analysis, Mann–Whitney U test, and Wilcoxon’s test were used to determine significance. A 95% confidence interval was considered significant and was defined as *p* < 0.05.

### Reagents

The source of the reagents and primer sequences was listed in Supplementary Table 1.

### Reporting summary

Further information on research design is available in the Nature Research Reporting Summary linked to this article.

## Supplementary information

Description of Supplementary Data

Supplementary Information

Supplementary Data 1

Supplementary Data 2

Reporting Summary

## Data Availability

Raw 16S rRNA gene sequence data for the feces microbiota were deposited in the NCBI Short Read Archive under BioProject Accession Number PRJNA326574. Microarray data Accession number GSE111111. The source data underlying plots presented in figures are shown in Supplementary Data 1. The data used for the L-kynurenine HPLC–MS/HPLC–MS/MS analyses and HPLC/MASS analyses of the gut contents are presented in Supplementary Data 2. The full blots are shown in Supplementary Fig. 11.
