# Supplementary material for: Lactobacillus maintains healthy gut mucosa by producing L-Ornithine
Source: Commun Biol. 2019 May 8;2:171. doi: 10.1038/s42003-019-0424-4 (PMC6506532; doi:10.1038/s42003-019-0424-4)
Supplement: Supplementary file 1 — Description of Supplementary Data [file 42003_2019_424_MOESM1_ESM.docx]

**Description of Additional Supplementary Files**

**File Name**: Supplementary Data 1

**Description**: Source data

**File Name**: Supplementary Data 2

**Description**: HPLC source data
