# Supplementary material for: Lactobacillus maintains healthy gut mucosa by producing L-Ornithine
Source: Commun Biol. 2019 May 8;2:171. doi: 10.1038/s42003-019-0424-4 (PMC6506532; doi:10.1038/s42003-019-0424-4)
Supplement: Supplementary file 2 — Supplementary Information [file 42003_2019_424_MOESM2_ESM.pdf]

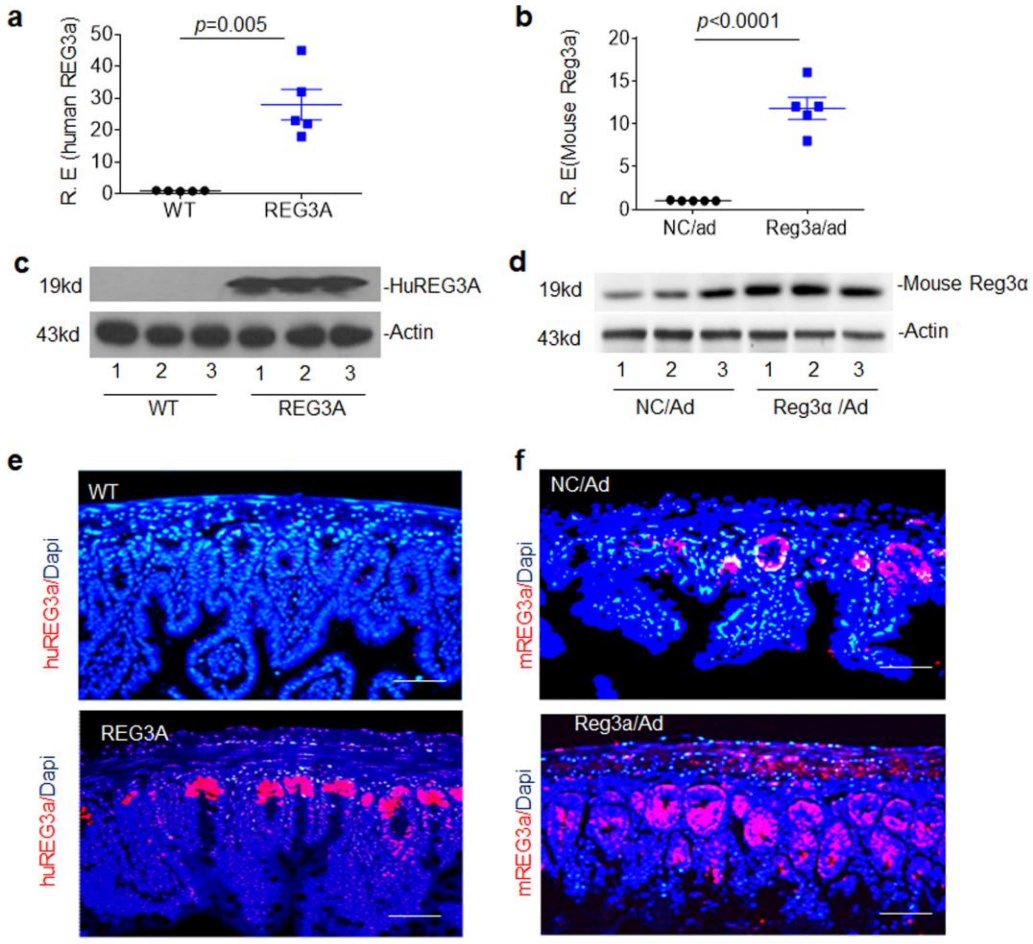

Supplementary Fig. 1. Expression of REG3〈 in human *REG3A* ^{tg} mice or Reg3〈/adenovirus injected mice. **a,** QRT-PCR of human REG3A in the ileum tissues from 6-8 week old male *wt* and human *REG3A* ^{tg} mice. **b,** QRT-PCR of mouse Reg3〈 in the ileum tissues from mice after ip injecting Reg3 〈/adenovirus (Reg3〈/Ad, 1×10^9viral particles/mouse) or control viral particles (NC/Ad, 1×10^9control viral particles/mouse) for two weeks. **c,** Immunoblot of human REG3A of ileum tissues from *wt* and human *REG3A* ^{tg} mice. **d,** Immunoblot of mouse Reg3〈 of the ileum tissues from mice with (Reg3〈/Ad) or without (NC/Ad) Reg3 〈 adenovirus injection. NC/Ad, empty adenovirus offered by company. **e,** Immunofluorescent staining of the ileum tissues from *wt* and human *REG3A* ^{tg} mice. **f,** Immunofluorescent staining of the ileum tissues from the mice with or without Reg3〈 adenovirus. Anti-human REG3A antibody was used in *wt* and human *REG3A* ^{tg} mice; whereas anti-mouse Reg3〈 antibody was used in the mice with (Reg3〈/Ad) or without (NC/Ad) Reg3 〈 /adenovirus injection. Scale bars=40 µm; Student's *t*-test in a and b, mean ±SD; NS, no significance; R. E., relative expression; Number in c and d indicates different individuals.

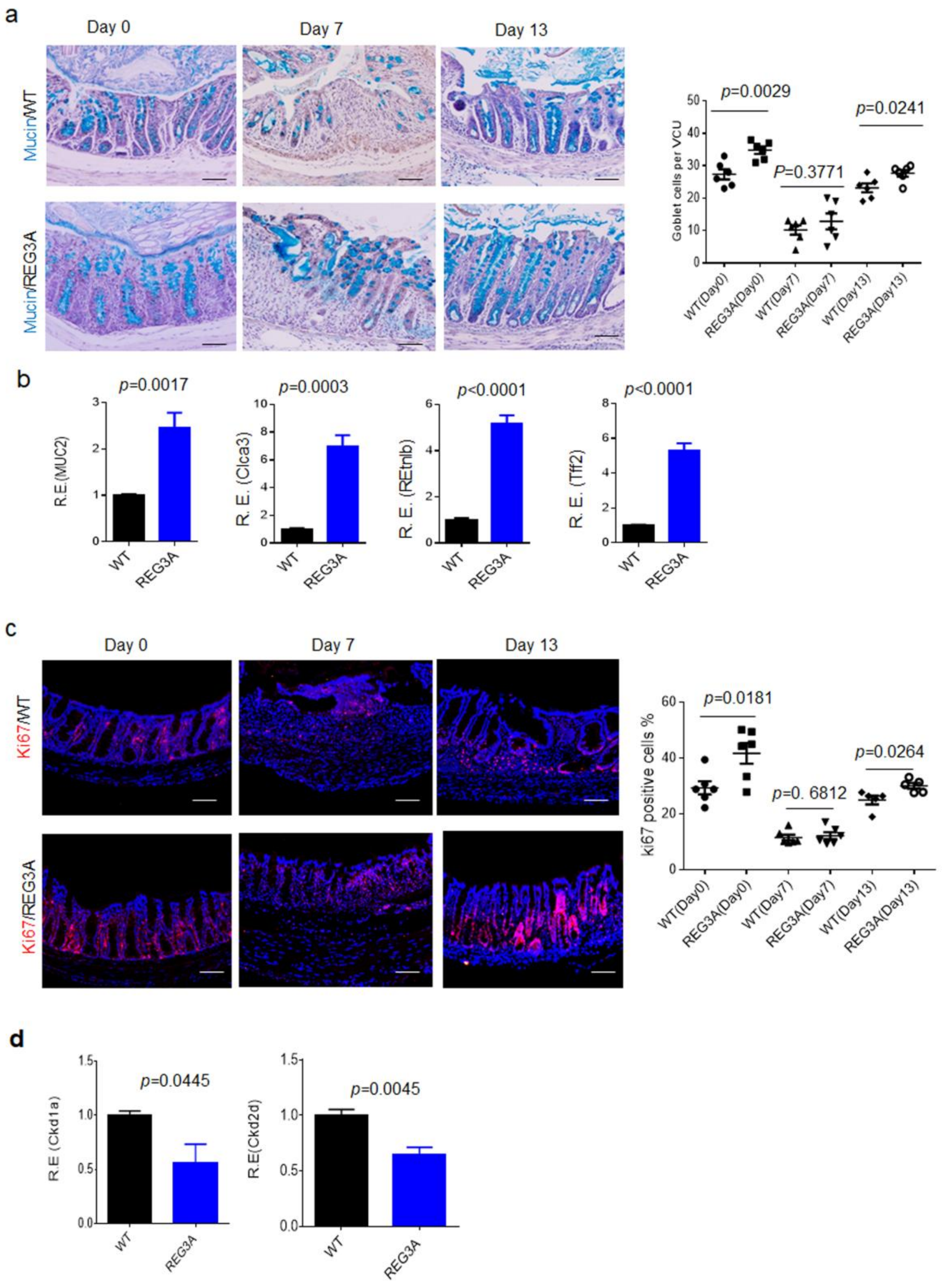

Supplementary Fig. 2. The mucus layer and proliferative index in the distal colons of REG3A transgenic mice. a, Staining of mucin in the proximal colon of human *REG3A* ^{tg} mice (REG3A) and control cohoused littermate *wt* mice at the indicated time (ten slides/mouse; n=6); VCU, villus-crypt units. **b,** QRT-PCR of mucin 2 (MUC2), Clca3, REtnlb and Tff2 in the colonic epithelial cells of human

REG3A ^{tg} and control cohoused littermate *wt* mice at day 13 (n=6); **c**, Staining of Ki67 cells in the colon (ten slides/mouse, n=6); The regions of interest were analyzed using ImageJ software, and detection of positive staining and cell number was performed with ImageJ software; **d**, QRT-PCR of Cdknla and Cdkn2d (n=6) in the colonic epithelial cells of human *REG3A* ^{tg} and control cohoused littermate *wt* mice at day 13 (n=6). These mice were treated using 2.5% DSS for 7 days from day 0, and then switched to regular drinking water. Scale bars=40 μm; Student's *t*-test in b and d; ANOVA plus post-Bonferroni analysis in a and c; mean ±SEM in a and c; NS, no significance; R. E, relative expression. Data are a representative of at least three independent experiments.

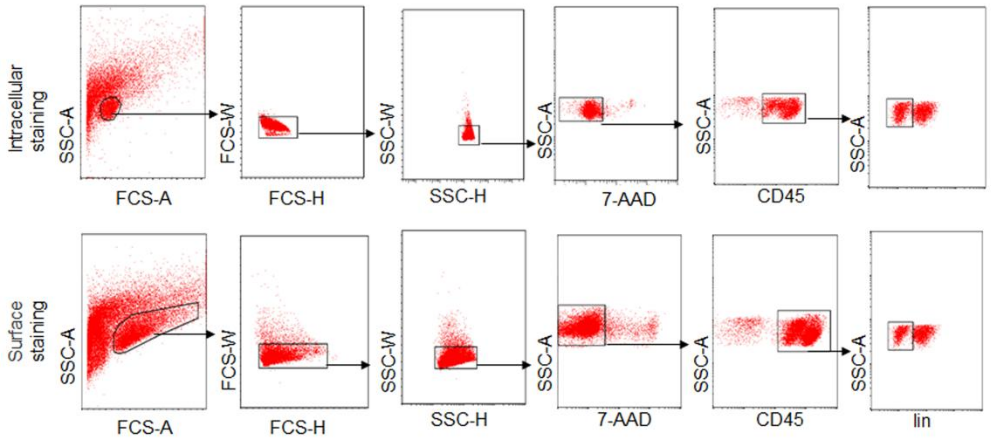

Supplementary Fig. 3. Representative FACS gating scheme for immune cell analyses in lamina propria (LP) tissues. After eliminating double cells by FCS-W and SSC-W, and dead cells by 7-AAD, we gated on lineage-negative cells for further analyses following initial gating on live CD45(+) cells.

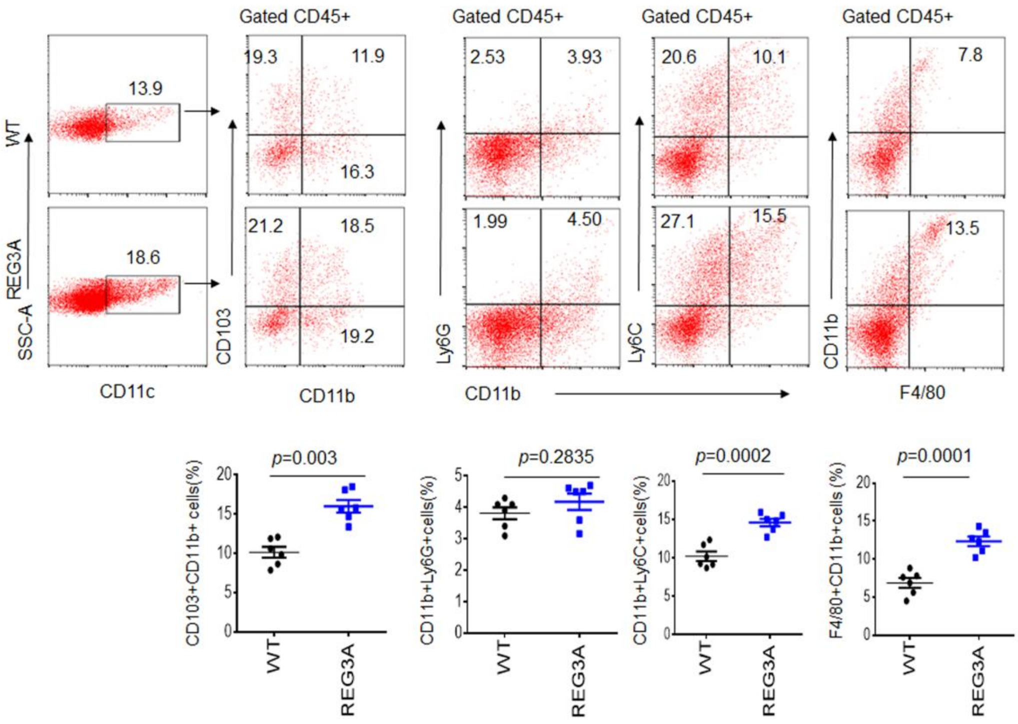

Supplementary Fig. 4. Immune cell populations in *REG3A* ^{tg} mice. Flow cytometry of CD11c(+) cells, CD11b(+)Ly6G(+), CD11b(+)Ly6C(+), F4/80(+)CD11b(+) and their subsets in the LP of ileum. Student's *t*-test was performed to compare the proportion of CD103(+)CD11b(+) , CD11b(+)Ly6G(+), CD11b(+)Ly6C(+), F4/80(+)CD11b(+) cells in *wt* and human *REG3A* ^{tg} (REG3*A*) mice (Mean ±SD, n=6). Numbers indicate the cellular proportion. Data are a representative of at least three independent experiments.

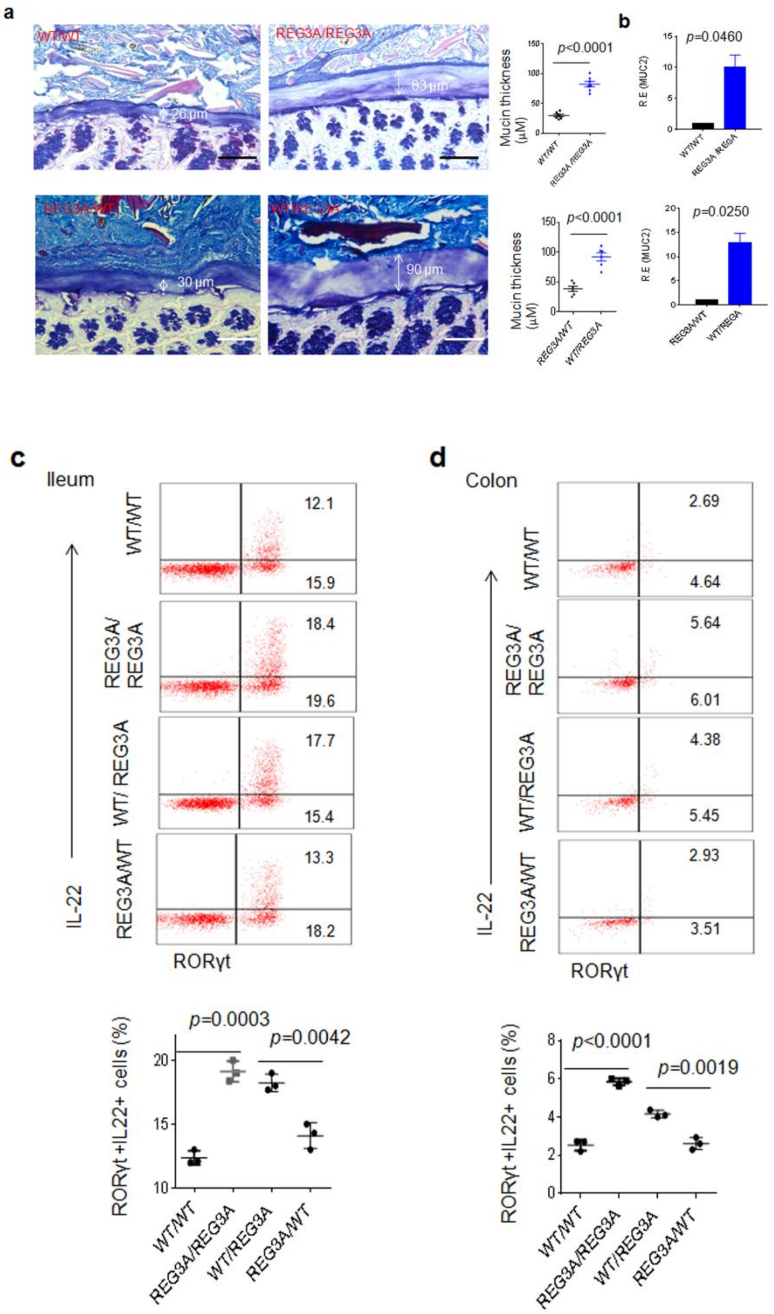

Supplementary Fig. 5. The transplantation of REG3A-shaped microbiota causes increased proportion of RORγt (+) IL-22(+) cells in small intestine and colon tissue. a, Mucus staining of colon tissues in WT/WT, REG3A/REG3A, WT/REG3A and REG3A/WT mice. **b,** QRT-PCR of MUC gene in the ileum and colon tissues of WT/WT, REG3A/REG3A, WT/REG3A and REG3A/WT mice; **c**, Flow cytometry of RORγt (+) IL-22(+) cells in the ileum and colon tissues of WT/WT, REG3A/REG3A, WT/REG3A and REG3A/WT mice. WT/WT, the feces of WT mice were transplanted

into pan-antibiotics treated mice; REG3A/REG3A, the feces of REG3A tg mice were transplanted into pan-antibiotics treated REG3A tg mice; WT/REG3A, the feces of REG3A tg mice were transplanted into pan-antibiotics treated WT mice; REG3A/WT, the feces of WT mice were transplanted into pan-antibiotics treated REG3A tg mice. Scale bars=40 μm. Student's *t*-test in a and b, ANOVA plus post-Bonferroni analysis in c and d. NS, no significance; R. E, relative expression.

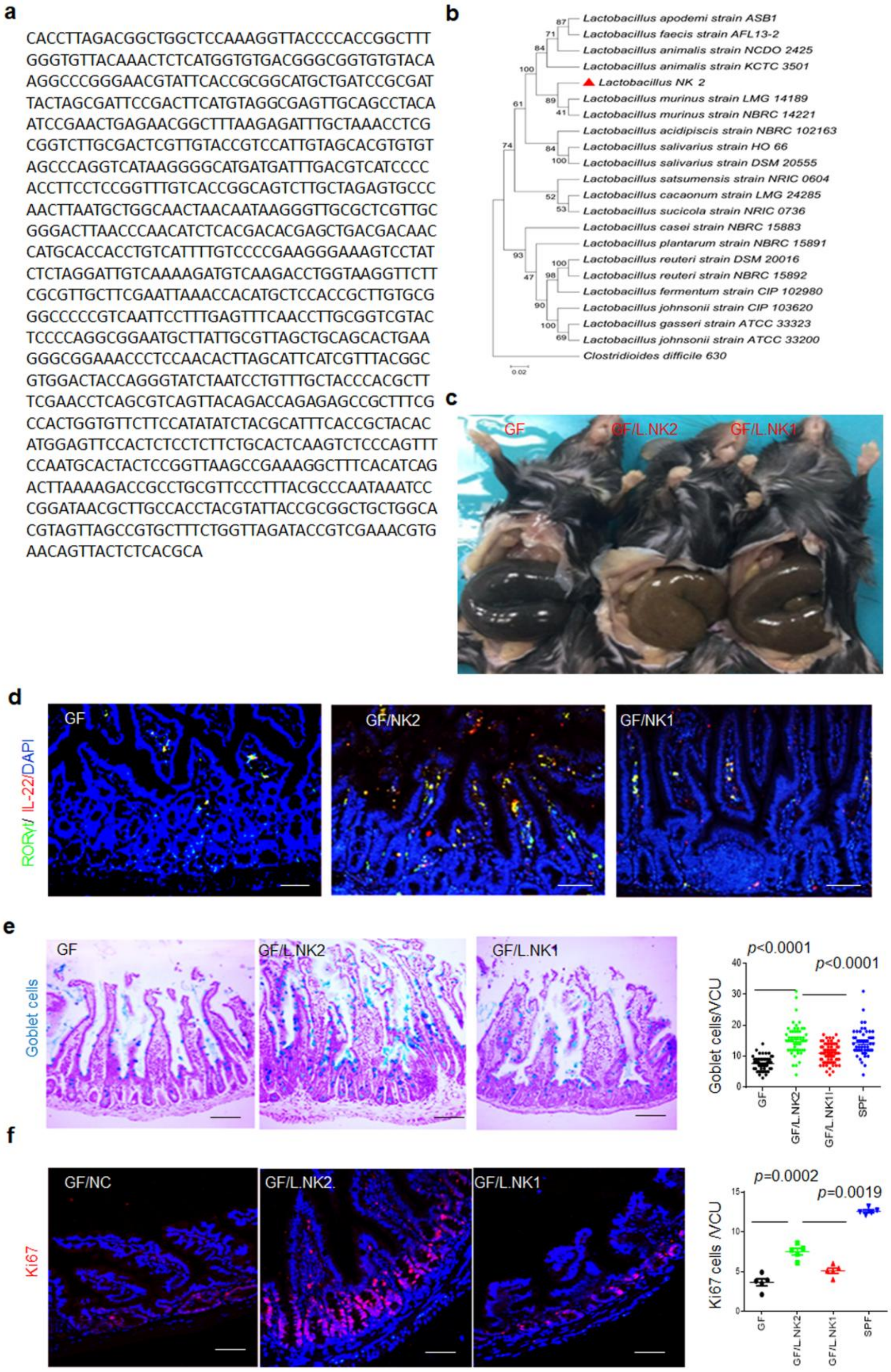

Supplementary Fig. 6. *Lactobacillus* promotes gut mucus layer formation in germ-free mice. a, Sequence of REG3A associated *lactobacillus*, which is named as

Lactobacillus NK.2(L.NK2). The 16s rRNAs from *lactobacillus* were extracted and sequenced by primers (F: 5'-AGAGTTTGATCATGGCTCAG-3'; R: 5'-TAGGGTTACCTTGTTACGACTT-3'). Fresh feacal samples were collected and diluted in 2 ml PBS solution, and cultured on Rogosa SL selective medium (Sigma-Aldrich) for *lactobacillus* enumeration, and then colonies were identified and purified using 16s rRNA sequence analyses. **b,** Homology of isolated *L. NK2* strain with other *lactobacilli.* The gut *lactobacillus* was selected and cultured in *lactobacillus* selected medium (Barebio, China). Phylogenetic tree shows the relationships among 16S rRNA sequences of *L. NK2* strain, including *lactobacillus* and species representing different lineages within genus *lactobacillus.* **c,** The representative caecum in germ-free (GF) mice with or without *L. NK2* or *L. NK1*colonization (n=5, male). **d,** Immunostaining of RORγt (+)IL-22(+) cells in the ileum of GF mice with or without *L.NK2* or *L.NK1* colonization. Representative images (n=6). **e,** Staining of goblet cells in the ileum of GF mice with or without *L.NK2* or *L.NK1* colonization. Ten slides/mouse, n=6. **f,** Staining of the Ki67 cells in the ileum of GF mice with or without *L.NK2* or *L.NK1* colonization. Ten slides/mouse, n=6. Scale bars=40 μm. ANOVA plus post-Bonferroni analysis in e and f; NS, no significance; R. E, relative expression.

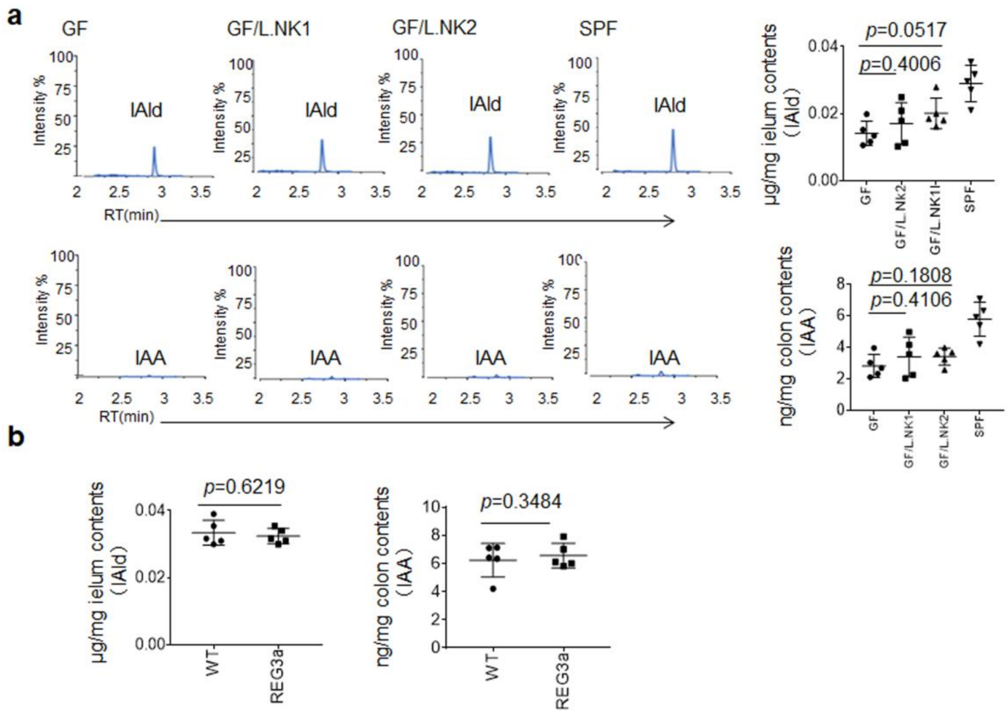

Supplementary Fig. 7. ***L. NK2*** **colonization does not change the levels of IAld and IAA in the ileum and colon of GF mice. a,** HPLC/MASS of IAld in the ileum and colon contents of GF mice with or without *L. NK2* or *L. NK1* colonization (n=5). **b,** HPLC/MASS of IAA in the ileum and colon contents of *wt* and *human REG3A* ^{tg} (REG3a) mice (n=5). ANOVA plus post-Bonferroni analysis in a; Student's *t*-test in **b**, mean ±SD. Data are a representative of three independent experiments.

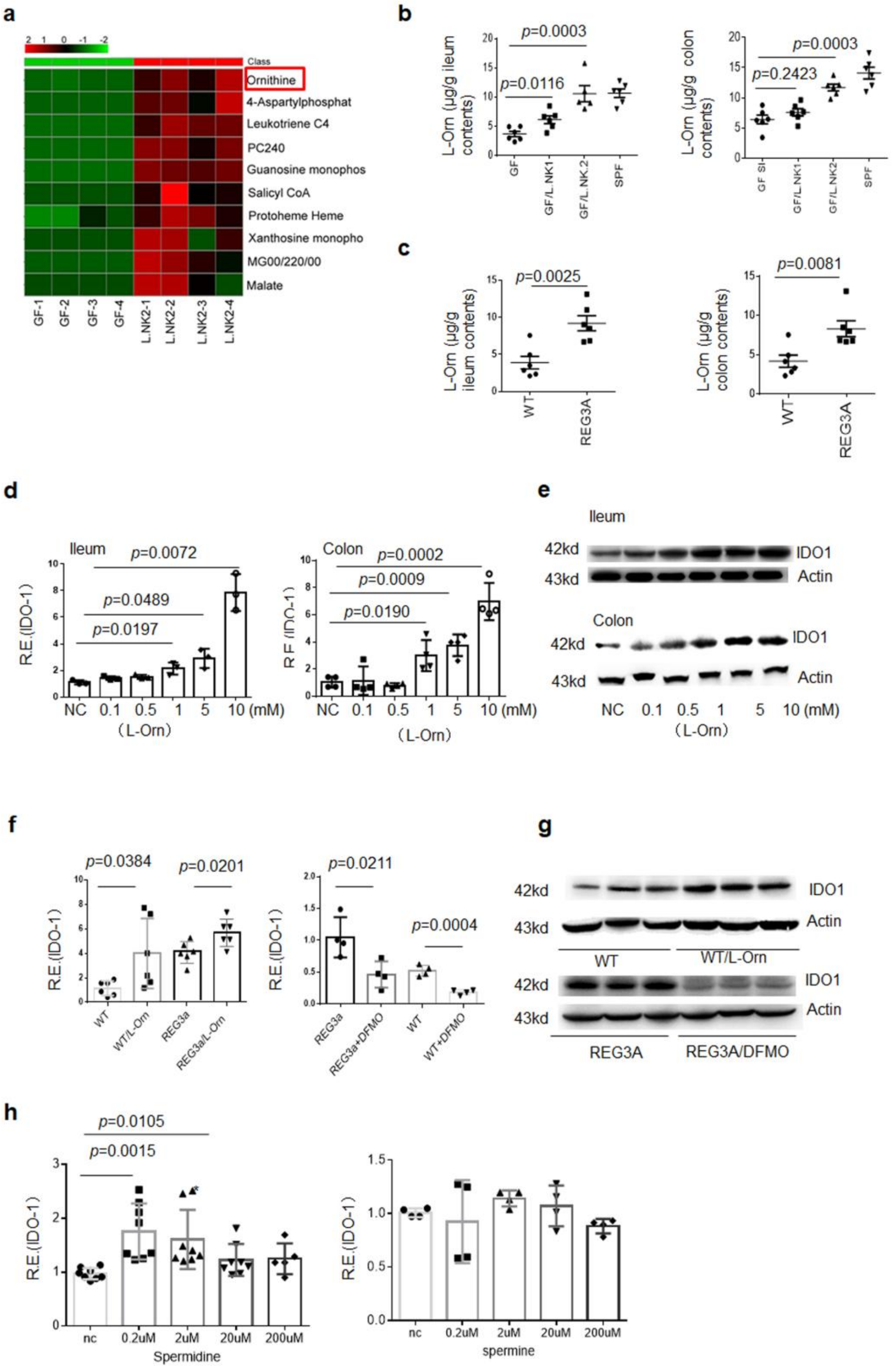
a
b
c
d
e
f
g
h
Class
Ornithine
4-Aspartylphosphat
Leukotriene C4
PC240
Guanosine monophos
Salicyl CoA
Protoheme Heme
Xanthosine monopho
MG00/220/00
Malate
GF-1
GF-2
GF-3
GF-4
L.NK2-1
L.NK2-2
L.NK2-3
L.NK2-4
L-Orn (µg/g ileum contents)
L-Orn (µg/g colon contents)
p=0.0003
p=0.0116
p=0.2423
GF
GF/L.NK1
GF/L.NK2
SPF
GF SI
L-Orn (µg/g ileum contents)
L-Orn (µg/g colon contents)
p=0.0025
p=0.0081
WT
REG3A
Ileum
Colon
R.E.(IDO-1)
p=0.0072
p=0.0489
p=0.0197
p=0.0002
p=0.0009
p=0.0190
NC
0.1
0.5
1
5
10 (mM)
（L-Orn）
42kd
43kd
IDO1
Actin
p=0.0384
p=0.0201
WT
WT/L-Orn
REG3a
REG3a/L-Orn
p=0.0211
p=0.0004
REG3a+DFMO
WT+DFMO
REG3A
REG3A/DFMO
p=0.0105
p=0.0015
nc
0.2uM
2uM
20uM
200uM
Spermidine
spermine

Supplementary Fig. 8. Gut tryptophan metabolism depends on L-Orn. a, HPLC/MASS of the gut contents in REG3〈 associated *lactobacillus* colonized GF mice. GF-1, GF-2, GF-3 and GF-4 indicated different GF individuals; L.NK2-1, L.NK2-2, L.NK2-3 and L.NK2-4 indicated different GF individuals infused by L. NK2 ($1{\cdot}10^9$/mouse). **b,** L-Orn analyses in the ileum and colon contents in REG3〈 associated *lactobacillus* (L. NK2) colonized GF mice. GF, germ free mice; GF/L.NK1, control *lactobacillus* infused GF mice; GF/L.NK2, REG3〈 associated *lactobacillus* infused mice; SPF, wt mice raised in specific pathogen-free environment. **c,** L-Orn analyses in the contents of the ileum and colon of *human REG3〈*^{tg} and their control littermate wt mice. **d** and **e,** QRT-PCR (d) and immunoblotting (e) of IDO1 in the ileum and colon epithelial cells after exposed to L-Orn. Isolated fresh ileum or colon were stimulated using different concentrations of L-Orn. IDO1 expression was analyzed after 12 hrs. **f** and **g,** QRT-PCR (f) and immunoblotting (g) of IDO1 in the ileum epithelial cells after L-Orn or L-Orn inhibitor DFMO (DFMO) administration. L-Ornithine and eflornithine monohydrochloride (DFMO) were dissolved in fresh water. The mean L-Orn consumption of mice was ~3.3 g/kg/d for 14 days; The mean DFMO consumption of mice was ~1.5 g/kg/d for 14 days. WT, wild type mice; WT/Orn, L-Orn fed *wt* mice; REG3A, human *REG3A*^{tg} mice; REG3A/DFMO, L-Orn inhibitor DFMO fed human *REG3A* ^{tg} mice. Mice fed with H_2O without L-Orn and DFMO were used as control. **h,** QRT-PCR of IDO1 in the ileum and colon epithelial cells after exposed to L-Orn. Isolated fresh ileum or colon were stimulated using different concentrations of spermidine and spermine. IDO1 expression was analyzed after 12 hrs. Student's *t*-test, mean ±SD in c ; ANOVA plus post-Bonferroni analysis in b, d, f and h. NS, no significance; R. E, relative expression. Data in per panel are a representative of at least three independent experiments.

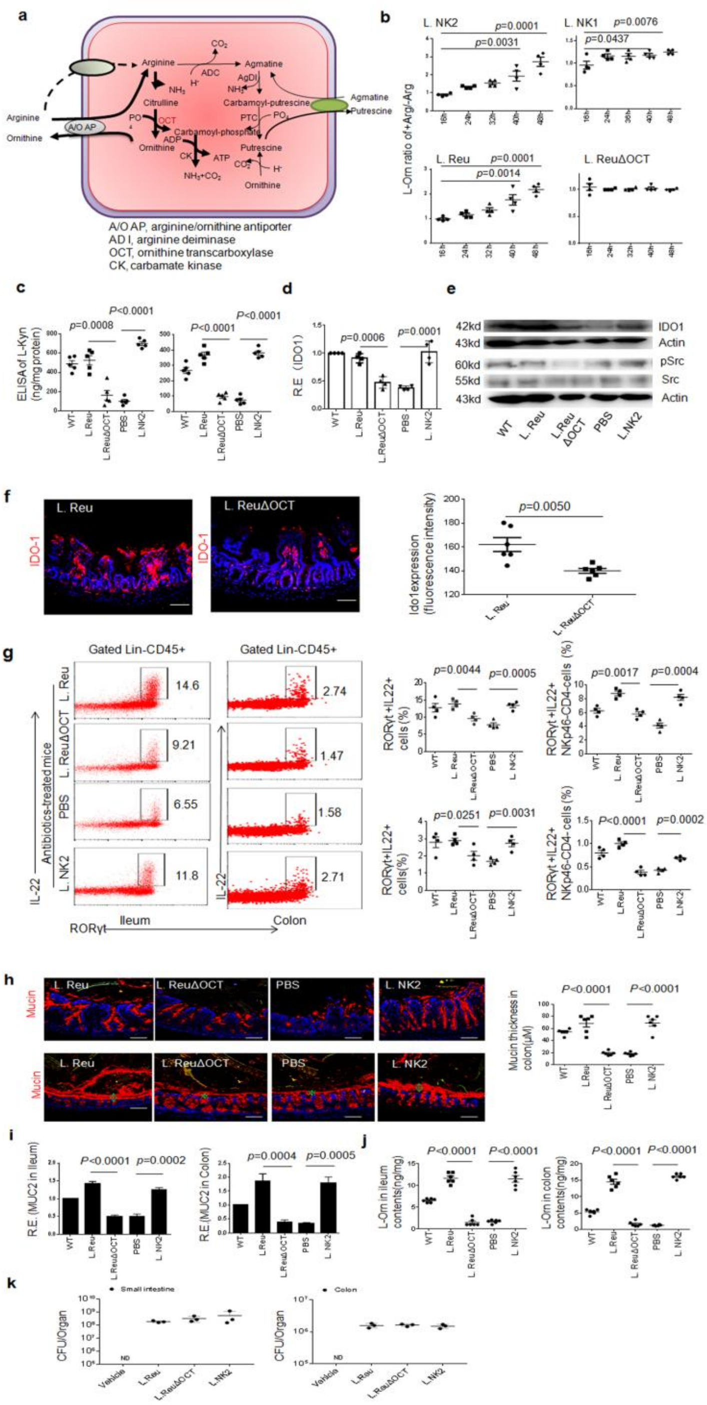

Supplementary Fig. 9. OCT deficiency impedes the role of lactobacillus. a, Map of arginine metabolism in lactobacillus. **b,** L-Orn analyses in the supernatants of different lactobacillus strain (L. NK2, L. NK1, L. Reuteri (L. Reu) and L. Reuteri/ΔOCT (L. ReuΔOCT) with and without arginine. The ratio of L-Orn with and without arginine was compared between 16 h and other time points. **c,** L-Kyn ELISA in the ileum and colon of mice. **d** and **e,** QRT-PCR (d) and immunoblotting (e) of IDO1 in the ileum epithelial cells of mice. **f,** Immunostaining of IDO1 in the ileum of

mice. **g,** Flow cytometry of CD45+RORγt (+) IL-22(+) cells and their subsets in the LP of ileum and colon of mice. **h,** Immunostaining of mucin in the ileum (upper) and colon (lower) of mice. **i,** QRT-PCR of mucin2 (Muc2) in the ileum (left) and colon (right) of mice. **j,** L-Orn analyses in the ileum (left) and colon (right) contents of mice. **k,** Clone forming units (CFU) of small intestine and colon in mice.

In **c-k,** L. Reuteri, L. Reuteri/ΔOCT, BPS and L. NK2 were respectively infused into antibiotics-treated mice for 7 days ($1 \cdot 10^9$ CFU/mouse); PBS, only PBS; WT, untreated control. Scale bars=40 µm; ANOVA plus post-Bonferroni analysis in b, c, d, g, h, i and j; Student's *t*-test, mean ±SD in f and k. NS, no significance; R. E, relative expression. Data are a representative of at least three independent experiments.

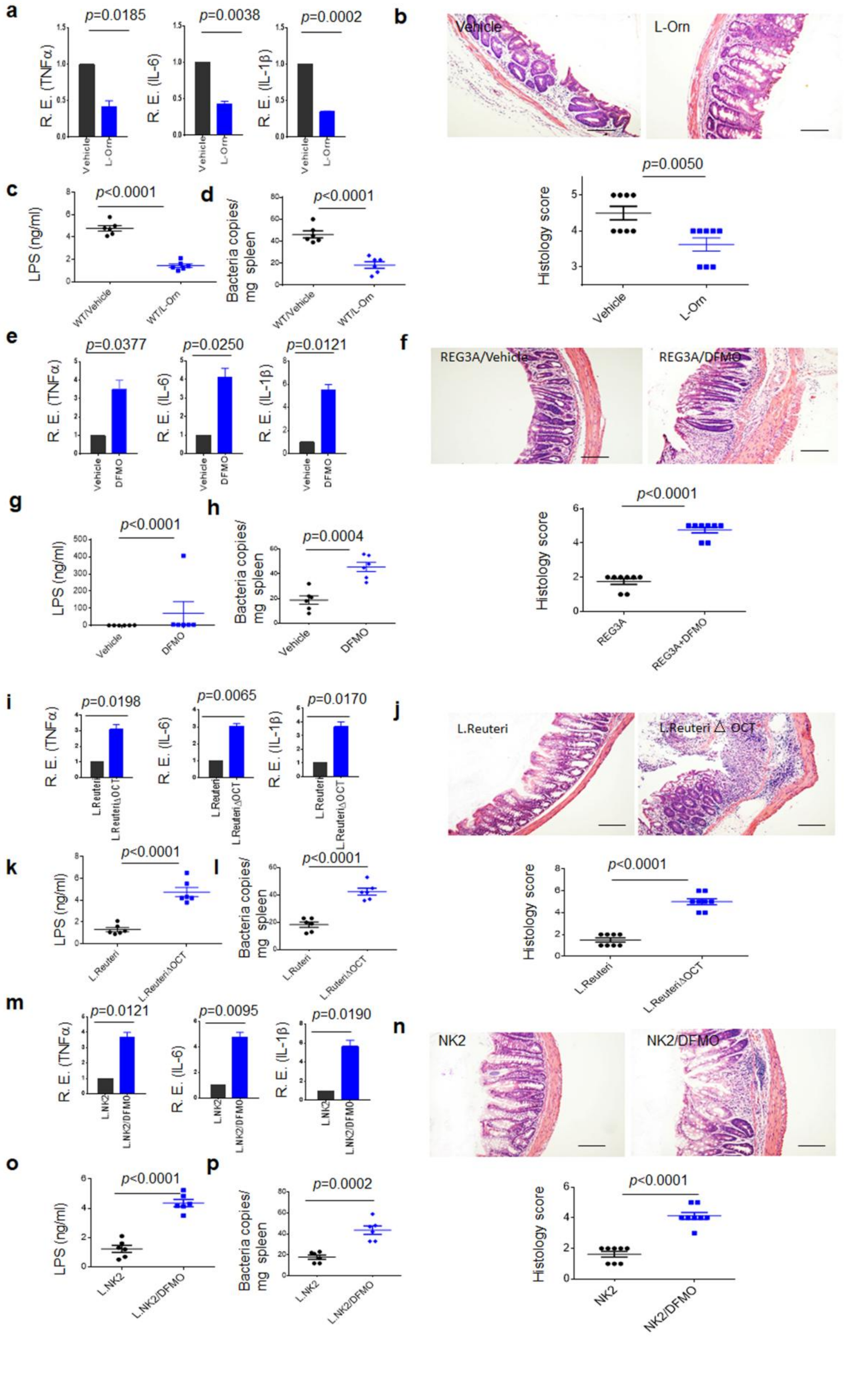
a
p=0.0185
p=0.0038
p=0.0002
R. E. (TNFα)
R. E. (IL-6)
R. E. (IL-1β)
Vehicle
L-Orn
b
Vehicle
L-Orn
p=0.0050
Histology score
c
p<0.0001
LPS (ng/ml)
WT/Vehicle
WT/L-Orn
d
p<0.0001
Bacteria copies/ mg spleen
e
p=0.0377
p=0.0250
p=0.0121
DFMO
f
REG3A/Vehicle
REG3A/DFMO
p<0.0001
REG3A
REG3A+DFMO
g
p<0.0001
h
p=0.0004
i
p=0.0198
p=0.0065
p=0.0170
L.Reuteri
L.Reuteri△OCT
j
L.Reuteri
L.Reuteri △ OCT
k
p<0.0001
l
p<0.0001
L.Ruteri
L.Ruteri△OCT
m
p=0.0121
p=0.0095
p=0.0190
L.NK2
L.NK2/DFMO
n
NK2
NK2/DFMO
p<0.0001
o
p<0.0001
p
p=0.0002

Supplementary Fig. 10. L-Orn promotes resistance to DSS mediated colitis. a, **e**, **i** and **m**, QRT-PCR of TNFα, IL-6 and IL1® in the colon tissues of *wt* mice with (L-Orn) or without (Vehicle) administration of L-Orn (n=6, male, a), *REG3A* ^{tg} *mice* with (REG3A/DFMO) or without (REG3A/Vehicle) administration of DFMO (n=6, male, e), mice after infusing *L. Reuteri* or *L. Reuteri/ΔOCT* mice (n=6, male, i) and mice after infusing *L. NK2 or L. NK2 with DFMO* mice (n=6, male, m). **b, f, j** and **n**, Hematoxylin/eosin staining and histological scores of distal colon samples in wt mice with (L-Orn) or without (Vehicle) administration of L-Orn (n=8, male, b), human *REG3A* ^{tg} *mice* with (REG3a/DFMO) or without (REG3a/Vehicle) administration of DFMO (n=8, male, f), mice after infusing *L. Reuteri* or *L. Reuteri/ΔOCT* mice (n=8, male, j) and mice after infusing *L. NK2 or L. NK2 with DFMO* (n=8, male, n). **c, g, k** and **o**, LPS in the peripheral sera of *wt* mice with (L-Orn) or without (Vehicle) administration of L-Orn (n=6, male, c), human *REG3A* ^{tg} *mice* with (REG3A/DFMO) or without (REG3A/Vehicle) administration of DFMO (n=6, male, g), mice after infusing *L. Reuteri* or *L. Reuteri/ΔOCT* mice (n=6, male, k) and mice after infusing *L. NK2 or L. NK2 with DFMO* mice (n=6, male, o). **d, h, l** and **p**, Bacterium numbers in the spleen in wt mice with (L-Orn) or without (Vehicle) administration of L-Orn (n=6, male, d), human *REG3A* ^{tg} *mice* with (REG3A/DFMO) or without (REG3A/Vehicle) administration of DFMO (n=6, male, h), mice after infusing *L. Reuteri* or *L. Reuteri/ΔOCT* mice (n=6, male, l) and mice after infusing *L. NK2 or L. NK2 with DFMO* (n=6, male, p). Scale bars=40 μm. Student's t-test, mean ±SD; NS, no significance; R. E., relative expression. Data are a representative of three independent experiments.

Figure4

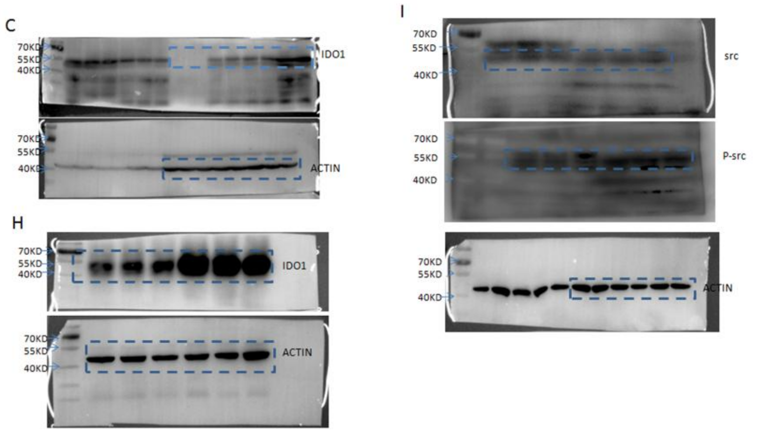

Figure S7

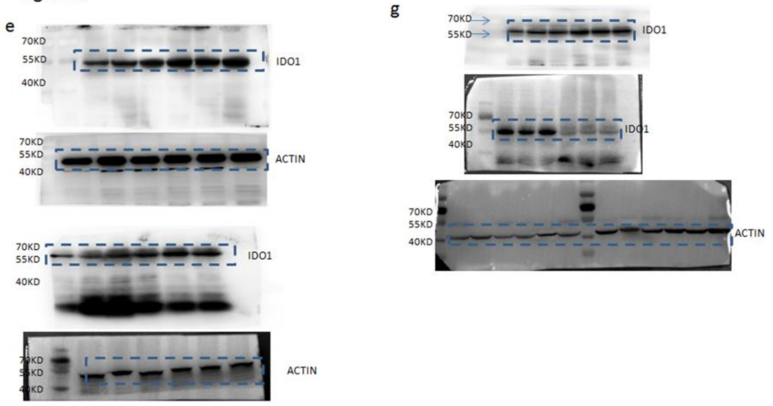

Figure S8

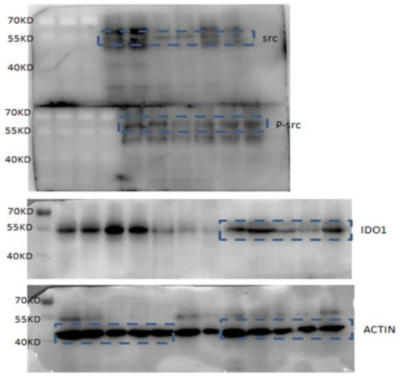

Supplementary Fig. 11. The full western blot in which the portions of blots and gels have been presented in the main paper.

Supplementary Table 1. The source of the reagents and primer sequences.

REAGENT or RESOURCE	SOURCE	IDENTIFIER
Antibodies for immunoblotting and immunostaining		
β-Actin (C4) mouse	Santa Cruz	Cat: sc-47778 RRID:AB_626632
REG3a (NP_002571.1) human	Abcam	Cat:ab95316 RRID:AB_10674667
REG3a (3x1R-7) mouse	Santa Cruz	Cat:sc-80319 RRID:AB_2178696
Mucin2 (H-300) mouse/human	Santa Cruz	Cat: sc-15334 RRID:AB_2146667
Lysozyme (w-20) mouse/human	Santa Cruz	Cat: sc-27956 RRID:AB_2138793
LGR5 (c-16) mouse/human	Santa Cruz	Cat: sc-68580 RRID:AB_2135160
IDO1(E-1) mouse/human	Santa Cruz	Cat: sc-376413 RRID:AB_11150511
Src (Clone 327) mouse	Abcam	Cat: ab16885 RRID:AB_443522
Src (phospho Y418) mouse/human	Abcam	Cat: ab4816 RRID:AB_304652
FITC-Goat Anti-Rat IgG(H+L)	Proteintech	Cat: SA00003-11
Alexa Fluor 488-Goat Anti-Mouse IgG(H+L)	Proteintech	Cat: SA00006-1
Alexa Fluor 594-Goat Anti-Rabbit IgG(H+L)	Proteintech	Cat: SA00006-4
Alexa Fluor 488-Goat Anti-Rabbit IgG(H+L)	Proteintech	Cat: SA00006-2
Antibodies for flow cytometry		
PerCP/Cy5.5-CD45 (30-F11) mouse	Biolegend	Cat:103132 RRID:AB_893340
BV421-CD4 (GK1.5) mouse	Biolegend	Cat:100438 RRID:AB_11203718
FITC-CD3 (17A2) mouse	eBioscience	Cat:11-0032-82 RRID:AB_2572431
FITC-CD19 (eBio1d3) mouse	Biolegend	Cat:115505 RRID:AB_313640
FITC-Gr1 (RB6-8C5) mouse	eBioscience	Cat:11-5931-85 RRID:AB_465315
PE-IL22 (Poly5164) mouse	Biolegend	Cat:516404 RRID:AB_2124255
APC-IL17 (eBio17B7) mouse	eBioscience	Cat:11-7177-81 RRID:AB_763581
FITC-F4/80 (BM8) mouse	Biolegend	Cat:123108 RRID:AB_893502
APC-RORγT (B2D) mouse	eBioscience	Cat:17-6981-82 RRID:AB_2573254
APC-CD11c (N418) mouse	Biolegend	Cat:117310 RRID:AB_313779
PE-CD103 (2E7) mouse	Biolegend	Cat:121405 RRID:AB_535948
PerCP/Cy5.5-CD11b (M1/70) mouse/human	Biolegend	Cat:101227 RRID:AB_893233
PE/Cy7-NKp46 (29A1.4) mouse	eBioscience	Cat:25-3351-80 RRID:AB_2573441
PE-Ly6G (1A8) mouse	BD Bioscience	Cat:551461 RRID:AB_394208
FITC-Ly6C (AL-21) mouse	BD Bioscience	Cat:553104 RRID:AB_394628
Alexa Fluor 700-CD45 (30-F11)	Biolegend	Cat: 103128 RRID:AB_493715
7-AAD	Biolegend	Cat. No. 34321X
Neutralizing antibody		

IL-22	Peprotech	Cat:500-P223 RRID:AB_1268324
Primers for Real-time PCR		
Murine GAPDH-Fs	BGI	5’-TCAACGGCACAGTCAAGG-3’
Murine GAPDH-Rs	BGI	5’-TACTCAGCACCGGCCTCA-3’
Murine MUC2	BGI	5’-ATGCCCACCTCCTCAAAGAC-3’
Murine MUC2	BGI	5’-GTAGTTTCCGTTGGAACAGTGAA-3’
Murine REG3a-Fs	BGI	5’-CAAGGCTTATCGCTCCCACT-3’
Murine REG3a-Rs	BGI	5’-ACGAGATGTCCTGAGGGTCT-3’
Human REG3a-Fs	BGI	5’-CATTGGTAACAGCTACTCATACGTCT-3’
Human REG3a-Rs	BGI	5’-CCTCAGAAATGCTGTGCTTCTCGAC-3’
Murine IFNg-Fs	BGI	5’-AACGCTACACACTGCATCTTGG-3’
Murine IFNg-Rs	BGI	5’-GACTTCAAAGAGTCTGAGG-3’
Murine TNFa-Fs	BGI	5’-GGTCTGGGCCATAGAACTGA-3’
Murine TNFa-Rs	BGI	5’-CAGCCTCTTCTCATTCCTGC-3’
Murine IL-6-Fs	BGI	5’-TCTGAAGGACTCTGGCTTTG-3’
Murine IL-6-Rs	BGI	5’-GATGGATGCTACCAAACTGGA-3’
Murine IL-1β-Fs	BGI	5’-GTGTCTTTCCCGTGGACCTT-3’
Murine IL-1β-Rs	BGI	5’-AATGGGAACGTCACACACCA-3’
Murine IL-22-Fs	BGI	5’-GCTCAGCTCCTGTCACATCA-3’
Murine IL-22-Rs	BGI	5’-CAGACGCAAGCATTTCTCAG-3’
Murine GM-CSF-Fs	BGI	5’-GCATGTAGAGGCCATCAAAGA-3’
Murine GM-CSF-Rs	BGI	5’-CGGGTCTGCACACATGTTA-3’
Murine IDO1-Fs	BGI	5’-CGGACTGAGAGGACACAGGTTAC-3’
Murine IDO1-Rs	BGI	5’-ACACATACGCCATGGTGATGTAC-3’
Murine Clca3-Fs	BGI	5’- AAGCAGCTGTGCTTCAGCAG -3’
Murine Clca3-Rs	BGI	5’- CAGATTGGACTTATCCACAG -3’
Murine Retnlb-Fs	BGI	5’- AAGCCTACACTGTGTTTCCTTTT -3’
Murine Retnlb-Rs	BGI	5’- GCTTCCTTGATCCTTTGATCCAC -3’
Murine Tff2 -Fs	BGI	5’- CTTGGTGTTTCCACCCACTT -3’
Murine Tff2 -Rs	BGI	5’- GGAAAAGCAGCAGTTTCGAC -3’
Murine Cdkn2d -Fs	BGI	5’-CGGTATCCACTATGCTTCTGGAA -3’
Murine Cdkn2d -Rs	BGI	5’-CCGCTGCGCCACTCA -3’
Murine Cadkn1a -Fs	BGI	5’-GTGGCCTTGTCGCTGTCT-3’
Murine Cadkn1a -Rs	BGI	5’-TTTTCTCTTGCAGAAGACCAATC -3’
Perimers for detection of bacteria		
Total Lactobacillus-Fs	BGI	5’-TGGATGCCTTGGCACTAGGA -3’

Total Lactobacillus-Rs	BGI	5’-AAATCTCCGGATCAAAGCTTACTTAT -3’
L. acidophilus-Fs	BGI	5’-GAAAGAGCCCAAACCAAGTGATT -3’
L. acidophilus-Rs	BGI	5’-CTTCCCAGATAATTCAACTATCGCTTA -3’
L.Reuteri-Fs	BGI	5’-ACCGAGAACACCGCGTTATTT -3’
L.Reuteri-Rs	BGI	5’-CATAACTTAACCTAAACAATCAAAGATTGTCT -3’
L.Johnsoni-Fs	BGI	5’-TCTTCCAATTTTTCGGCAGT -3’
L.Johnsoni-Rs	BGI	5’-CAGTGGGAGCTACAGAAGCA -3’
L.Plantorum-Fs	BGI	5’-CTCTGGTATTGATTGGTGCTTGCAT -3’
L.Plantorum-Rs	BGI	5’-GTTCGCCACTCACTCAAATGTAAA -3’
L.Murinus-Fs	BGI	5’-AGCTAGTTGGTGGGGTAAAG -3’
L.Murinus-Rs	BGI	5’-TAGGATTGTCAAAAGATGTC -3’
L.NK1-Fs	BGI	5’-CATCCAGTGCAAACCTAAGAG -3’
L.NK1-Rs	BGI	5’-GATCCGCTTGCCTTCGCA -3’
L.NK2-Fs	BGI	5’-AGCTAGTTGGTGAGGTAAAG -3’
L.NK2-Rs	BGI	5’-TAGGATTGTCAGAAGATGTC -3’
Bacteroides Phylum-Fs	BGI	5’-GAGAGGAAGGTCCCCCAC -3’
Bacteroides Phylum-Rs	BGI	5’-CGCTACTTGGCTGGTTCAG -3’
Firmicutes Phylum-Fs	BGI	5’-GCTGCTAATACCGCATGATATGTC -3’
Firmicutes Phylum-Rs	BGI	5’-CAGACGCGAGTCCATCTCAGA -3’
Perimers for deletion of OCT		
Left-Fs	BGI	5’-ATGGCAATCGTTTCAGCAGTGCGAGATTATTAAGAC-3’
Left-Rs	BGI	5’-CAATTTTATTAAAGTTCATCAAGTACTATAATAGGC-3’
Cat-Fs	BGI	5’-GCCTATTATAGTACTTGATGAACTTTAATAAAATTG-3’
Cat-Rs	BGI	5’-CATGGCAAATGCCTCCTAATTATAAAAGCCAGTCATTAG-3’
Right-Fs	BGI	5’-CTAATGACTGGCTTTTATAATTAGGAGGCATTTGCCATG-3’
Right-Rs	BGI	5’-CGAATTACGAATTTTTCTTAGTTATCACGAATAAC-3’
36e-Fs	BGI	5’-GTTATTCGTGATAACTAAGAAAAATTCGTAATTCG-3’
36e-R	BGI	5’-GTCTTAATAATCTCGCACTGCTGAAACGATTGCCAT-3’
Perimers for analysis of deletion targets		
OCT-Fs	BGI	5’-ATGGCTTTTAATTTACGTA-3’
OCT-Rs	BGI	5’-TTAGTTTTGTTCACCCAAAGT-3’

OCT-up	BGI	5'-CTTATTGACTTTGGCTTG-3'
OCT-dw	BGI	5'-CTGTCCAATATGGGAATG-3'
Perimers for generation and identifition of Human REG3atg mice		
M13F	BGI	5'-GCCAGGGTTTTCCCAGTCACGA-3'
HD5-REG3A-tR	BGI	5'-GTAGGGTATGATGTGACGTTTG-3'
HD5-tR	BGI	5'-CAGCATGGTGGTACATGCCT-3'
CDS-tF	BGI	5'-GGCAACATATGCCCATATGC-3'
REG3A -tF3	BGI	5'-GAGCCCAATGGAGAAGGTTGG-3'
REG3A -tR3	BGI	5'-GTCCTTCCGAGTGAGAGACAC-3'
Probe		
16S rRNA (Eub338)	PNA BIO	Cy3-GCTGCCTCCCGTAGGAGT
Lactobacillus 16S rRNA (Lac663)	PNA BIO	FAM-O-ACATGGAGTTCCACT
Other reagents		
REG3α/Ad	ABM	Cat: 176974A
LPS ELISA KIT	Elabscience	Cat: E-EL-0025C
Mouse Ornithine ELISA KIT	JiangSu Meibiao	Cat: MB-5610A
Mouse KYN ELISA KIT	ImmuSmol	Cat: BA-E-2200
FITC-dextran (40,000kD)	Sigma	Cat: 53379
AB-PAS staining kits	Leagene	Cat: DG0007
Ampicillin	Sigma	Cat: BP021
Vancomycine	Sigma	Cat: V2002
Neomycin sulfate	Sigma	Cat: N6386
Metronidazole	Sigma	Cat: M3761
L-Ornithine	Sigma	Cat: O2375
L-Arginine	Sigma	Cat: W381918
Standard kynurenine	meilunbio	Cat: MB5637
L-kyn sulfate	Sigma	Cat: K3750
Eflornithine (DFMO)	MCE	Cat: HY-B0744B
PP2	MCE	Cat: HY-13805
rIL-23	Gibco	Cat: PHC9324
High-fat diet	Research Diets	Cat: D12492
Rogosa SL selective medium	Sigma	Cat: R1148
MRS	3M US	Cat: BP0275500
Trizol	Life Technologies	Cat: 15596026
QIAquick PCR Purification Kit	Qiagen	Cat:28104
QuantiTect SYBR Green PCR Master Mix	Qiagen	Cat:208052

FBS	Gibco	Cat:10099141
Collagenase IV	Sigma	Cat: C5138
Dnase I	Solarbio	Cat: D8071
DMEM	Gibco	Cat:11965118
HBSS	Gibco	Cat:14170161
Pecoll	Solarbio	Cat: P8370
Cell stimulation cocktail	ebioscience	Cat: 00-4975-03
Foxp3 fix/perm buffer	Biolegend	Cat: 421403
PMA	Sigma	Cat: 79346
GolgiStop	BD Biosciences	Cat: 554724
Permeabilization Buffer	eBioscience	Cat: 00-8333-56
Alexa Fluor™ 488 Tyramide Reagent	Life Technologies	Cat: T20948
Ki67antibody	Elabscience	Cat: E-AB-31869
