# Supplementary material for: Lactobacillus maintains healthy gut mucosa by producing L-Ornithine
Source: Commun Biol. 2019 May 8;2:171. doi: 10.1038/s42003-019-0424-4 (PMC6506532; doi:10.1038/s42003-019-0424-4)
Supplement: Supplementary file 5 — Reporting Summary [file 42003_2019_424_MOESM5_ESM.pdf]

nature research

Corresponding author(s): Rongcun Yang

Last updated by author(s): Apr 5, 2019

Reporting Summary

Nature Research wishes to improve the reproducibility of the work that we publish. This form provides structure for consistency and transparency in reporting. For further information on Nature Research policies, see Authors & Referees and the Editorial Policy Checklist.

Statistics

For all statistical analyses, confirm that the following items are present in the figure legend, table legend, main text, or Methods section.

n/a	Confirmed	
☐	☒	The exact sample size (*n*) for each experimental group/condition, given as a discrete number and unit of measurement
☐	☒	A statement on whether measurements were taken from distinct samples or whether the same sample was measured repeatedly
☐	☒	The statistical test(s) used AND whether they are one- or two-sided<br>*Only common tests should be described solely by name; describe more complex techniques in the Methods section.*
☐	☒	A description of all covariates tested
☐	☒	A description of any assumptions or corrections, such as tests of normality and adjustment for multiple comparisons
☐	☒	A full description of the statistical parameters including central tendency (e.g. means) or other basic estimates (e.g. regression coefficient) AND variation (e.g. standard deviation) or associated estimates of uncertainty (e.g. confidence intervals)
☐	☒	For null hypothesis testing, the test statistic (e.g. *F*, *t*, *r*) with confidence intervals, effect sizes, degrees of freedom and *P* value noted<br>*Give P values as exact values whenever suitable.*
☒	☐	For Bayesian analysis, information on the choice of priors and Markov chain Monte Carlo settings
☐	☒	For hierarchical and complex designs, identification of the appropriate level for tests and full reporting of outcomes
☒	☐	Estimates of effect sizes (e.g. Cohen's *d*, Pearson's *r*), indicating how they were calculated

Our web collection on statistics for biologists contains articles on many of the points above.

Software and code

Policy information about availability of computer code

Data collection

1)Gut microbiota were analyzed by Majorbio Biotechnology Company (Shanghai, China) using primers that target to the V3-V4 regions of 16S rRNA;
2)Expression of coding mRNA and miRNA were analyzed by Beijing Capitalbio Technology Co., Ltd. using Affymetrix 3 IVT Genechip arrays; The quality of isolated RNA samples was evaluated with an Agilent Bioanalyzer 2100 (Agilent technologies) and the purified RNA was quantified using a NanoDrop ND-2000 spectrophotometer (Thermo Fisher).
3)Single-cell suspensions of Peyer's patches (PP) and spleen of mice were analyzed by flow cytometry (BD FACS Calibur or BD LSRFortessa);
4) Hematoxylin/eosin (H&E) staining and Immunostaining were analyzed by Leica DMI 6000B and/or Zeiss LSM 700 confocal microscope.
5) Immunostaining of mucus layers and localization of bacteria by Fluorescent in situ hybridization by Leica DMI 6000B and/or Zeiss LSM 700 confocal microscope.
6)HPLC-MS and HPLC-MS/MS analyses were performed by ProfLeader Biotechnology Company (Shanghai, China) through a Waters Xevo-TQXS system or water acquity UPLL system.
7) ELISAs were analysed using TECAN infinite M200PRO.

Data analysis	1) For Gut microbiota analyses, Operational Taxonomic Unit (OTU) analysis was performed as follows: Sequences were processed (trimmed) using the Mothur software; For each clustering, Morisita-Horn dissimilarity was used to compute a sample distance matrix from the initial count matrix, and the distance matrix was subsequently used to generate a hierarchical clustering using Ward's minimum variance method;<br>2) Expression of coding mRNA and miRNA were analyzed using Cluster 3.0 and MAS 3.0;<br>3) Flow cytometry data were analyzed by FlowJo-vio<br>4) Hematoxylin/eosin (H&E) staining and Immunostaining were analyzed by Image J.<br>5) Graphbad Prism was used in other analyses such as qRT-PCR, ELISA etc..<br>5) Immunostaining of mucus layers and localization of bacteria by Fluorescent in situ hybridization by Image J.<br>6) HPLC-MS and HPLC-MS/MS analyses of L-kynurenine were performed by ProfLeader Biotechnology Company (Shanghai, China) through Analyst (V.1.5.2), Masslynx 4.1 and SIMCA-P13.0.

For manuscripts utilizing custom algorithms or software that are central to the research but not yet described in published literature, software must be made available to editors/reviewers. We strongly encourage code deposition in a community repository (e.g. GitHub). See the Nature Research guidelines for submitting code & software for further information.

Data

Policy information about availability of data

All manuscripts must include a data availability statement. This statement should provide the following information, where applicable:

- Accession codes, unique identifiers, or web links for publicly available datasets
- A list of figures that have associated raw data
- A description of any restrictions on data availability

1)Raw 16S rRNA gene sequence data for the feces microbiota were deposited in the NCBI Short Read Archive under BioProject Accession Number PRJNA326574, Which is related to Figure 3A and 2B,
2)Microarray data: https://www.ncbi.nlm.nih.gov/geo/query/acc.cgi?acc=GSE111111, which is related to Figure 4A.
3) Data of flow cytometry , histological and immunostaining, immunostaining of mucus layers and localization of bacteria by Fluorescent in situ hybridization, L-kynurenine HPLC-MS and HPLC-MS/MS analyses, HPLC/MASS analyses of gut contents, qRT-PCR, immunoblotting and ELISA, which are related to Figure 1 to Figure 6, and supplementary data may be found in 317 Lab, Nankai university school of medicine, Nankai university.

Field-specific reporting

Please select the one below that is the best fit for your research. If you are not sure, read the appropriate sections before making your selection.

☒ Life sciences ☐ Behavioural & social sciences ☐ Ecological, evolutionary & environmental sciences

For a reference copy of the document with all sections, see nature.com/documents/nr-reporting-summary-flat.pdf

Life sciences study design

All studies must disclose on these points even when the disclosure is negative.

Sample size	1) For fluorescence in situ hybridization of 16s rRNA and immunostaining of mucin, ten slides/mouse; n=6;<br>2) For flow cytometry and qRT-PCR, n=6;<br>3) For 16SrRNA, microarray and HPLC/Mass, n=6;<br>4)For DSS mediated colitis model, n=18
Data exclusions	No data explusions
Replication	Except for 16SrRNA, microarray and HPLC/Mass, other experiments were repeated at least three times.
Randomization	For comparision between WT and huREG3[illegible] tg mice, huREG3[illegible] tg mice (REG3[illegible]) and control cohoused littermate wt mice were used;<br>The age- and gender- matched mice were randomized into different groups.
Blinding	The investigators were blinded to group allocation during data collection and/or analyses.

Reporting for specific materials, systems and methods

We require information from authors about some types of materials, experimental systems and methods used in many studies. Here, indicate whether each material, system or method listed is relevant to your study. If you are not sure if a list item applies to your research, read the appropriate section before selecting a response.

Materials & experimental systems

n/a	Involved in the study
☐	☒ Antibodies
☒	☐ Eukaryotic cell lines
☒	☐ Palaeontology
☐	☒ Animals and other organisms
☒	☐ Human research participants
☒	☐ Clinical data

Methods

n/a	Involved in the study
☒	☐ ChIP-seq
☐	☒ Flow cytometry
☒	☐ MRI-based neuroimaging

Antibodies

Antibodies used

Antibodies used in this study
β-Actin (C4) mouse Santa Cruz Cat: sc-47778 RRID:AB_626632
REG3a (NP_002571.1) human Abcam Cat:ab95316 RRID:AB_10674667
REG3a (3x1R-7) mouse Santa Cruz Cat:sc-80319 RRID:AB_2178696
Mucin2 (H-300) mouse/human Santa Cruz Cat: sc-15334 RRID:AB_2146667
Lysozyme (w-20) mouse/human Santa Cruz Cat: sc-27956 RRID:AB_2138793
LGR5 (c-16) mouse/human Santa Cruz Cat: sc-68580 RRID:AB_2135160
IDO1(E-1) mouse/human Santa Cruz Cat: sc-376413 RRID:AB_11150511
Src (Clone 327) mouse Abcam Cat: ab16885 RRID:AB_443522
Src (phospho Y418) mouse/human Abcam Cat: ab4816 RRID:AB_304652
FITC-Goat Anti-Rat IgG(H+L) Proteintech Cat: SA00003-11
Alexa Fluor 488-Goat Anti-Mouse IgG(H+L) Proteintech Cat: SA00006-1
Alexa Fluor 594-Goat Anti-Rabbit IgG(H+L) Proteintech Cat: SA00006-4
Alexa Fluor 488-Goat Anti-Rabbit IgG(H+L) Proteintech Cat: SA00006-2
Antibodies for flow cytometry
PerCP/Cy5.5-CD45 (30-F11) mouse Biolegend Cat:103132 RRID:AB_893340
BV421-CD4 (GK1.5) mouse Biolegend Cat:100438 RRID:AB_11203718
FITC-CD3 (17A2) mouse eBioscience Cat:11-0032-82 RRID:AB_2572431
FITC-CD19 (eBio1d3) mouse Biolegend Cat:115505 RRID:AB_313640
FITC-Gr1 (RB6-8C5) mouse eBioscience Cat:11-5931-85 RRID:AB_465315
PE-IL22 (Poly5164) mouse Biolegend Cat:516404 RRID:AB_2124255
APC-IL17 (eBio17B7) mouse eBioscience Cat:11-7177-81 RRID:AB_763581
FITC-F4/80 (BM8) mouse Biolegend Cat:123108 RRID:AB_893502
APC-RORγT (B2D) mouse eBioscience Cat:17-6981-82 RRID:AB_2573254
APC-CD11c (N418) mouse Biolegend Cat:117310 RRID:AB_313779
PE-CD103 (2E7) mouse Biolegend Cat:121405 RRID:AB_535948
PerCP/Cy5.5-CD11b (M1/70) mouse/human Biolegend Cat:101227 RRID:AB_893233
PE/Cy7-NKp46 (29A1.4) mouse eBioscience Cat:25-3351-80 RRID:AB_2573441
PE-Ly6G (1A8) mouse BD Bioscience Cat:551461 RRID:AB_394208
FITC-Ly6C (AL-21) mouse BD Bioscience Cat:553104 RRID:AB_394628
Alexa Fluor 700-CD45 （30-F11） Biolegend Cat: 103128 RRID:AB_493715
7-AAD Biolegend Cat. No. 34321X
Neutralizing antibody
IL-22 Peprotech Cat:500-P223 RRID:AB_1268324

Validation

Antibodies for Validation
β-Actin (C4) , specific for mouse β-Actin ;
REG3a (NP_002571.1), specific for human and mouse REG3a;
REG3a (3x1R-7) , specific for mouse REG3a;
Mucin2 (H-300), specific for mouse/human mucin2;
Lysozyme (w-20) , specific for mouse/human lysozyme;
LGR5 (c-16), specific for mouse/human LGR5;
IDO1(E-1) , specific for mouse/human IDO1;
Src (Clone 327) , specific for mouse Src;
Src (phospho Y418), sepcific for mouse/human Src (phospho Y418);
FITC-Goat Anti-Rat IgG(H+L); specific for Rat IgG(H+L) ;
Alexa Fluor 488-Goat Anti-Mouse IgG(H+L) ; specific for Mouse IgG(H+L);
Alexa Fluor 594-Goat Anti-Rabbit IgG(H+L); specific for Rabbit IgG(H+L);
Alexa Fluor 488-Goat Anti-Rabbit IgG(H+L) ; specific for abbit IgG(H+L) .
Antibodies for flow cytometry
PerCP/Cy5.5-CD45 (30-F11) , specific for mouse CD45;
BV421-CD4 (GK1.5), specific for mouse CD4;
FITC-CD3 (17A2) , specific for mouse CD3;
FITC-CD19 (eBio1d3), specific for mouse CD19 ;
FITC-Gr1 (RB6-8C5), specific for mouse Gr1;
PE-IL22 (Poly5164) , specific for mouse IL-22;
APC-IL17 (eBio17B7) , specific for mouse IL-17;
FITC-F4/80 (BM8), specific for mouse F4/80;

APC-RORγT (B2D), specific for mouse RORγT ;
APC-CD11c (N418) , specific for mouse CD11C;
PE-CD103 (2E7) , specific for mouse CD103;
PerCP/Cy5.5-CD11b (M1/70) , specific for mouse/human CD11b;
PE/Cy7-NKp46 (29A1.4), specific for mouse NKp46;
PE-Ly6G (1A8) , specific for mouse Ly6G;
FITC-Ly6C (AL-21), specific for mouse Ly6C;
Alexa Fluor 700-CD45 （30-F11） ;
7-AAD Biolegend Cat. No. 34321X;
Neutralizing antibody
IL-22, specific for mouse IL-22 ;

Animals and other organisms

Policy information about studies involving animals; ARRIVE guidelines recommended for reporting animal research

Laboratory animals	Human REG3? transgenic mice (huREG3? tg) mice, C57BL/6; IDO-1-/- mice, C57BL/6 ; germ-free (GF) mice, C57BL/6; Wild-type mouse, C57BL/6.
Wild animals	No Wild animals were used in this study.
Field-collected samples	1) All experimental litters were bred and maintained under specific pathogen-free （SPF） conditions in the Animal Center of Nankai University.<br>2)C57BL/6 germ-free (GF) mice were generated by Shanghai SLAC Laboratory Animal Co. LID. All experiments in GF mice were performed in Shanghai SLAC Laboratory Animal Co. LID.
Ethics oversight	All procedures were conducted according to the Institutional Animal Care and Use Committee of the Model Animal Research Center. Animal experiments were approved by the Institute’s Animal Ethics Committee of Nankai University.

Note that full information on the approval of the study protocol must also be provided in the manuscript.

Flow Cytometry

Plots

Confirm that:

- [x] The axis labels state the marker and fluorochrome used (e.g. CD4-FITC).
- [x] The axis scales are clearly visible. Include numbers along axes only for bottom left plot of group (a 'group' is an analysis of identical markers).
- [x] All plots are contour plots with outliers or pseudocolor plots.
- [x] A numerical value for number of cells or percentage (with statistics) is provided.

Methodology

Sample preparation	1)Single-cell suspensions of Peyer’s patches (PP) and spleen of mice were prepared by mashing in a cell strainer (70 mm), stained and analyzed by flow cytometry;<br>2)For the staining of lamina propria (LP) lymphocytes, colon or small intestine were isolated, cleaned by shaking in ice-cold PBS four times before tissue was cut into 1 cm pieces. The epithelial cells were removed by incubating the tissue in HBSS with 2 mM EDTA for 30 min at 37 ℃ with shaking. The LP cells were isolated by incubating the tissues in digestion buffer (DMEM, 5% fetal bovine serum, 1 mg/ ml Collagenase IV and DNase I for 40 min. The digested tissues were then filtered through a 40-mm filter. Cells were resuspended in 10 ml of the 40% fraction of a 40: 80 Percoll gradient and overlaid on 5 ml of the 80% fraction in a 15 ml Falcon tube. LP cells were collected at the interphase of the Percoll gradient, washed and resuspended in medium, and then stained and analyzed by flow cytometry. Dead cells were eliminated through 7-AAD staining.<br>3)For intracellular staining, the cells were cultured and stimulated for 6 hrs with 50ng/ml phorbol 12-myristate 13-acetate and 1 μg/ml ionomycin (Sigma) in the presence of GolgiStop. After incubation for 6 hrs, cells were washed in PBS, and then fixed in Cytofix/Cytoperm, permeabilized with Perm/Wash buffer, and stained with FITC-, PE-, APC- APC/cy7-, PerCP/Cy5.5- or PE/cy7-conjugated antibodies. Meanwhile, dead cells were eliminated through 7-AAD staining.<br>4)For IL-22 production, cells were stimulated directly ex vivo by incubating for 6 hr with 20 ng/ ml rIL-23 in the presence of cell stimulation cocktail for the final 3 hr of culture. Cells were fixed and permeabilized by using Foxp3 fix/perm buffer set, as described by manufacturers and stained with IL-22 and RORγt antibodies.
Instrument	BD FACS Calibur
Software	FlowJo. Vio
Cell population abundance	undo this
Gating strategy	1) For RORgammaT(+)IL-22(+)cells, CD4+IL-22+, CD4+IL-17+, IL-17+IL-22+, NKp46 and CD4+ cells<br>after FCS-A and SSC-A as well as FCS-W and SSC-W, the 7-AAD （-） CD45 （+） Lin （-） alive simple cell population was gated to analyze these cell populations.

2)For CD11b+CD103+, CD11b+Ly6G+, CD11b+Ly6C+ and F4/80 and CD11b+ cells, after FCS-A and SSC-A as well as FCS-W and SSC-W, the 7-AAD（-） CD45（+） alive simple cell population were gated to analyze these cell populations.

☒ Tick this box to confirm that a figure exemplifying the gating strategy is provided in the Supplementary Information.
